# A Narrative Review of Metallomic Studies: Revealing the Toxic Metal Burden in Tobacco Smokers

**DOI:** 10.3390/ijms262311617

**Published:** 2025-11-30

**Authors:** Wojciech Flieger, Magdalena Stankiewicz, Eliasz Dzierżyński, Piotr Gawlik, Łukasz Pietrzyk, Mirosław Łańcut, Filip Walczak, Anna Szymkiewicz, Jolanta Flieger

**Affiliations:** 1 Department of Plastic Surgery, St. John’s Cancer Center, Jaczewskiego 7, 20-090 Lublin, Poland; idziarzhynski@cozl.pl (E.D.); pgawlik@cozl.pl (P.G.); 2 Institute of Health Sciences, John Paul II Catholic University of Lublin, Konstantynów 1 H, 20-708 Lublin, Poland; lukasz.pietrzyk@kul.pl; 3 Department of Infectious Diseases, Medical University of Lublin, 20-059 Lublin, Poland; magdastankiewicz77@gmail.com; 4 Experimental Research Center, John Paul II Catholic University of Lublin, Konstantynów 1 H, 20-708 Lublin, Poland; miroslaw.lancut@kul.pl; 5 Holycross Cancer Center, Department of Genetic Engineering, Artwinskiego 3, 25-734 Kielce, Poland; filip.walczak@onkol.kielce.pl (F.W.); anna.szymkiewicz@onkol.kielce.pl (A.S.); 6 Department of Analytical Chemistry, Medical University of Lublin, Chodźki 4a (Collegium Pharmaceuticum), 20-093 Lublin, Poland

**Keywords:** toxic metals, elemental homeostasis, bioaccumulation, elemental analysis, tobacco smokers

## Abstract

Metallomics, which studies the role of metals in biological processes, is crucial for understanding the impact of elements on human health. It requires an integrated approach combining quantitative and functional methods, supported by advanced analytical techniques. A comprehensive understanding of metallomics considers the accumulation, speciation, and distribution of metals. In recent decades, an increasing number of studies have focused on determining metal levels in human tissues, particularly in the context of chronic diseases and developmental disorders. Levels of macro- and microelements, both essential and toxic, play a fundamental role in both physiological and pathological processes. Given the increasing health risks associated with addictions such as smoking, understanding the mechanisms of toxicity based on metallomic studies is crucial. This literature review synthesizes current advances in analytical techniques used to determine trace elements in biological samples, the accumulation of toxic metals, and the disruption of element homeostasis in tobacco smokers. The aim of this study is to identify key risks from tobacco-related metal exposure, thereby providing a deeper understanding of the long-term health consequences. The obtained results may constitute the basis for future directions of metallomic research.

## 1. Introduction

Excessive accumulation of metals in tissues is primarily caused by exposure to a polluted anthropogenic environment and toxic dietary contaminants [1]. Psychoactive substance abuse is also a source of exposure to many organic and inorganic toxins [2,3]. With the development of civilization, we are observing an increase in the incidence of addictions (including alcoholism, drug addiction, and nicotine addiction), which are considered in the context of psychological or physical causes, taking into account the negative effects of poisoning the body. Despite growing public awareness and evidence of the harmful effects of smoking, nicotine addiction and tobacco smoking continue to cause disease and premature death [4]. 2025 will mark over 20 years since the entry into force of the World Health Organization’s (WHO) Framework Convention on Tobacco Control (FCTC), signed in Geneva on 21 May 2003 [5]. Currently, approximately 28.8% of adults in Poland smoke tobacco, which is higher than the European Union (EU) average of 25.3%. In comparison, in Sweden, only 5–8% of the population smokes [6]. On 16 January 2024, the WHO published the “WHO Global Report on Trends in Tobacco Prevalence 2000–2030,” which emphasized that although smoking rates have declined in most countries, there are still 1.25 billion adult tobacco users worldwide, and the number of deaths caused by tobacco smoking will begin to decline in the next few years [7]. According to the WHO, one person currently dies every 4 s from smoking. Tobacco cigarette consumption and the number of smokers are high, particularly in countries with lower economic status. Given the risk of addiction and its health consequences, there is an urgent need for research to determine the effects of exposure, understand the pathogenic mechanisms and their relationship to the accumulation of toxins present in tobacco smoke, develop strategies for preventing intoxication, and identify potential diagnostic parameters and new therapeutic approaches.

An important aspect of cigarette smoking is the disruption of homeostasis, micronutrient and trace element status, and the accumulation of toxic elements. The first study on the determination of selected metals, i.e., iron (Fe), copper (Cu), zinc (Zn), manganese (Mn), calcium (Ca), and magnesium (Mg), in the tissues of nicotine addicts dates back to 1991 [8]. This study was continued in 2001, measuring selenium (Se), Zn, Cu, and Fe in the plasma of tobacco smokers [9]. In both cases, quantitative determinations were performed using atomic absorption spectrometry (AAS). Regarding counteracting the consequences of smoking, no trace elements have demonstrated efficacy as a diagnostic instrument or prospective supplement/medication.

The literature review aimed to gather current research on the risk of toxic metal accumulation in tobacco smokers, which would allow for the identification of the greatest risks associated with cigarette smoking, which, with long-term exposure, can lead to serious and permanent health effects. An initial search of the PubMed, Web of Science, and Scopus databases from 2000 to 2025 was conducted based on the article title and abstract to gather information on the latest results and trends in metallomic research related to tobacco smoking. Duplicates were excluded. The lead authors decided on the inclusion/exclusion of articles based on their novelty and the relevance of the study findings. Articles were included if full text was available. Ultimately, 474 articles were included, sorted qualitatively according to the subsection topics. The summary includes biological sample preparation, the latest analytical techniques, comparative studies using different tissues and body fluids in smoker/nonsmoker groups, and the mechanisms of toxicity of the most frequently detected toxic metals in smokers: lead (Pb), cadmium (Cd), nickel (Ni). The final section discusses challenges that may determine trends in future metallomic research.

## 2. Introduction to Metallomics

R.J.P. Williams was the first to use the term “metalome” to describe the specific pathways of metal distribution in the body [10]. However, Hiroki Haraguchi, a professor at Nagoya University, is considered the pioneer and founder of metallomics, initiating the integration of various scientific fields to assess the role of metals in complex biological systems [11,12].

According to the International Union of Pure and Applied Chemistry (IUPAC), metallomics is defined as “the totality of metal and metalloid species present in a biological system, defined in terms of their identity and/or quantity” [13].

Metallomics can be divided into structural, functional, and quantitative metallomics. Therefore, there is a subset of metallomics that concerns the qualitative identification of individual elements (qualitative metallomics) and their concentration (quantitative metallomics). In turn, the assessment of element content in biological materials, related to function, falls within the scope of functional metallomics. It should be emphasized that metallomics is a holistic approach aimed at understanding the role of metals in biology and medicine [14,15,16,17,18]. Since quantitative aspects are usually the basis for metallomic interpretation, there is a need for multidisciplinary research using a set of modern tools [19]. Quantitative metallomic studies must meet the criteria of specificity, sensitivity and be flexible enough to cover a wide range of concentrations, the complex chemistry of the sample matrix, and account for the dynamics of possible transformations.

Currently, elemental quantification has significantly expanded its capabilities, primarily thanks to the development of advanced analytical techniques, primarily inductively coupled plasma mass spectrometry (ICP-MS), which enables simultaneous qualitative and quantitative determination of many chemical elements present at trace and ultratrace levels [20,21]. In turn, the coupling of spectroscopic and microscopic techniques has led to the development of in situ metallomic imaging techniques, which open up new perspectives for metal speciation, with the possibility of multi-element tracking and generating isotopically specific maps of element distribution in complex biological matrices, complemented by the identification of valence states and the entire environment, i.e., interactions and functional connections of metals with genes, proteins (metalloproteomes), metabolites, and other endogenous or bioinduced biomolecules, such as organic acids, sugars, or DNA fragments in the cell [22,23,24]. Analytical techniques as tools of structural metallomics, thanks to its high sensitivity (10–100 ppq) and spatial resolution at the level of 1 submicron, enable the transition from the atomic level to systemic processes [25]. Analytical techniques used for the quantitative determination of elements in biological materials should be adapted to the needs of the study. Currently available techniques differ significantly in incomparable detection limits (DL), influences of the organic matrix, or various types of interferences [26].

Defining the metalome allows us to determine: (i) the distribution of metals within individual compartments; (ii) the concentration of individual metals; and (iii) the type of complexed bioligands. Different tissues have varying requirements, leading to an uneven distribution of metal ions at the (sub)cellular level, within individual systems, and throughout the body. For instance, approximately 90% of the total iron (Fe) in the body is found in the blood as a component of hemoglobin. Zinc (Zn) is a cellular ion present in blood plasma and other bodily fluids, while calcium (Ca) is the primary component of bones. The distribution of these elements within cells is also uneven. Iron accumulates in the mitochondria, except in red blood cells; calcium is primarily found in the cytosol; and zinc is located in the cell nucleus. Of note, the content of so-called non-essential elements in tissues, such as rubidium (Rb), strontium (Sr), and titanium (Ti), is noteworthy. The Mayo Clinic states that there is no evidence that titanium is an essential element. Quantitatively, titanium is a (ultra)trace element, typically found in sub-ppb concentrations, although higher levels have been detected in artificial joints, prostheses, implants, etc. Their functions are not yet fully understood, and it is unknown to what extent they disrupt the functions of essential elements, particularly through binding to proteins in metalloproteomes. In the human body, metal levels, both macro- and micronutrients, are subject to dynamic changes and are critical for health [27]. The mechanisms ensuring both systemic homeostasis and cellular balance are complex and specific to individual metal ions [28]. Many proteins responsible for the acquisition and distribution of chemical elements participate in this process [29]. Accessory proteins that ensure the binding of the correct metal are called metallochaperones. The term “chaperone” was introduced in the late 20th century by Tom O’Halloran, who studied the role of the copper chaperone in Cu,Zn-superoxide dismutase (SOD) [30]. Under physiological conditions, specific metalloproteins bind to specific metals. Biometals, such as manganese (Mn), molybdenum (Mo), zinc (Zn), iron (Fe), and copper (Cu), are essential components of metalloproteins. They act as cofactors necessary for various metabolic processes. Cobalt serves as a cofactor primarily in the structure of cobalamin (vitamin B12), which is vital for enzymes like methionine synthase and methylmalonyl-CoA mutase. If proteins bind to the wrong metal, their function can be inhibited. Therefore, controlling metal availability is crucial, as it ensures the proper selectivity of metal ion binding to proteins. This is particularly important for Fe and Zn, which can occupy the same binding sites. It is known that approximately 30% of proteins are metallomes, i.e., transport proteins in the cytoplasm [31,32,33]. To ensure the appropriate amount of metals necessary for proper cellular function, living organisms possess homeostatic mechanisms, utilizing a range of acquisition and storage proteins, transporters, metal chaperones, and metal-sensing transcriptional regulators [34]. In this way, the organism can avoid metal overload and the associated toxic effects.

The “biology of iron” is well understood. In the body, Fe occurs as Fe^2+^ and Fe^3+^ ions with varying reactivity and complex stability. It should be noted that iron research has been ongoing since the 18th century, when Menghini first identified Fe in human blood [35,36]. However, the essential role of Zn for humans was not proven until the 1960s. There is evidence suggesting that bromine (Br) may be an essential element for life, although this idea is not yet widely accepted. A study conducted in 2014 by Scott McCall and colleagues described bromine as an essential trace element for the formation of collagen scaffolds, which are important for tissue development and architecture [37]. Currently, the prevailing view is that there are more than 20 elements essential for human life, including (macro)minerals: hydrogen (H), carbon (C), nitrogen (N), oxygen (O), sulfur (S), phosphorus (P), chlorine (Cl), sodium (Na), potassium (K), Mg, Ca, as well as trace elements such as Fe, Zn, Mn, Cu, Co, Mo, Se, iodine (I). In fact, since the 1960s, the concept of indispensability has been modified with respect to mineral elements. Some trace elements were not considered essential, such as boron (B), silicon (Si), chromium (Cr), and nickel (Ni), but it has now been recognized that they reduce the risk of chronic diseases, and their deficiency in the diet adversely affects biological functions. Other examples include Cr or fluorine (F), which supports dental health; however, this is not considered to be essential for life [38,39]. Elements such as Fe and Zn occur at the gram level (2–5 g/70 kg human), hence some authors classify them as minerals [40,41,42], in contrast to trace elements, which occur in quantities two (Cu), three (Mn), or four orders of magnitude smaller (Mo, Cr, Co). However, in body fluids, reference ranges (or values) for various elements established for non-diseased people are established for diagnostic purposes. For example, the Mayo Clinic has defined reference ranges for serum zinc from 66 to 106 mcg/dL in adults. For serum iron, the reference ranges for men are 50–150 mcg/dL, and for women: 35–145 mcg/dL. In turn, Skalnaya and Skalny, in their monograph on biometals [43], considered macroelements to be those elements whose concentration in the body exceeds 0.01% (O, C, H, N, Ca, P, K, Na, S, Cl, Mg). The absolute mass of macroelements per 70 kg of a human ranges from a few grams (Mg) to over forty kilograms (O). The next group, known as trace elements, ranges from 0.00001% to 0.01%. (Fe, Zn, F, Sr, Mo, Cu, Br, silicon (Si), caesium (Cs), I, Mn, aluminium (Al), Pb, Cd, boron (B), Rb), which translates to mass amounts from hundreds of milligrams to several grams. Ultratrace elements have contents of less than 0.000001% (Se, Co, vanadium (V), Cr, arsenic (As), Ni, lithium (Li), barium (Ba), Ti, silver (Ag), tin (Sn), beryllium (Be), gallium (Ga), germanium (Ge), mercury (Hg), scandium (Sc), zirconium (Zr), bismuth (Bi), antimony (Sb), uranium (U), thorium (Th), rhodium (Rh)), and their absolute mass is expressed in milligrams (mg) or micrograms (µg). Among the essential trace elements mentioned above, such as Co, Cr, Cu, Fe, Mn, Mo, Zn, and Se, the human body also accumulates toxic metals that do not perform any physiological function, such as Al, As, Hg, Pb, Be, Ni, Cd, thallium (Tl), etc. There is no universal definition of trace elements, as their classification can vary depending on the perspective—nutritional (macro and micronutrients), environmental, or within human biology, clinical chemistry, and toxicology. The CLSI C38 standard document titled “Control of Preexamination Variation in Trace Element Determinations,” 2nd edition, published in 1997, categorizes elements based on their concentration in the body. It defines trace elements as those with body content ranging from 0.01 to 100 µg/g (10 µg/L to 10^4^ µg/L), and ultratrace elements as those with body content of less than 0.01 µg/g (10 µg/L) [44].

It should be noted that many non-essential elements, considered toxic and referred to as “heavy metals,” such as Ba, As, Al, Cd, Pb, and Hg, are often present at dangerously high levels, depending on the duration and intensity of exposure. According to the IUPAC, the term “heavy metals” associated with and used in the context of pollution and potential toxicity or ecotoxicity should not be used. The report’s authors suggest that characterization should be based on chemical classification based on the periodic table of elements and predicted toxic effects [45].

**Figure 1:**
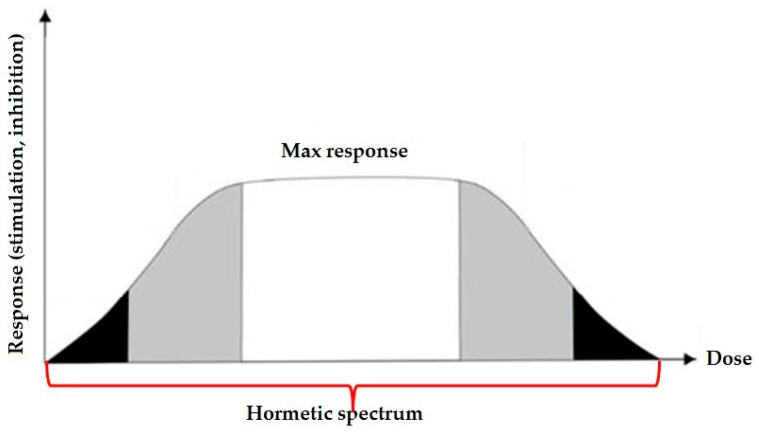
Schematic dose–response relationship illustrating the concept of hormesis.

Complex interactions occur between essential, non-essential, and even toxic elements. For example, an overload or deficiency of one element can affect the others. For example, excessive zinc supplementation can result in copper deficiency [41]. Despite the existence of compensatory systems, chronic exposure to environmental factors and/or reduced excretion capacity leads to the accumulation of toxic metals in tissues depending on the absorbed dose, the route of exposure, and the duration of exposure (acute or chronic) [42,43]. Metals, like other agents, more often exhibit a hormetic dose-response relationship. This type of dependence, characterized by beneficial effects at low doses and adverse effects at high doses, is a general concept within the field of medicine and toxicology. To report hormetic responses, many other terms can be used, including the Arndt-Schulz Law, biphasic dose response, U-shaped dose response, preconditioning/adaptive response, overcompensation responses, rebound effect, repeat bout effect, and steeling effect, among others [46]. There are many examples in scientific literature of hormetic dose-response curves. For example, one of the trace elements, selenium (Se), which is essential for the proper function of selenoproteins, is toxic at high doses, causing a condition called selenosis. Iron is essential for red blood cells and serves an important function in other cell types, but excessive iron intake can cause oxidative damage to tissues. The hormetic response for Fe and Cu has been well established, and dysregulation of iron and copper homeostasis is involved in a wide range of diseases, including neurodegenerative disorders such as Parkinson’s disease [47]. This hormetic dose-response is a perfect illustration of the famous maxim of Paracelsus, which states that “*Dosis sola facit venenum*”, i.e., only the dose makes the poison (Figure 1) [48,49].

There are many examples confirming this principle [50,51,52]. For example, Mn deficiency caused by a poor diet leads to the development of the so-called metabolic syndrome, diabetes, etc. [53,54]. On the other hand, Mn toxicity, manifested by manganism, is observed in association with the consumption of contaminated water with high Mn content or other repeated occupational and nutritional excessive exposure to Mn. Zinc (Zn^2+^) participates as a component of metalloproteins in many enzymatic catalytic processes. Zn distribution within the cell is tightly controlled to avoid zinc ion overload, which causes protein misfolding and aggregation. The mechanisms of intracellular Zn metalloregulation are described in detail in the review by Maret and Li [55]. Much is known about chromium Cr(III), which is an essential metal, competitive with cations such as Fe(III), in contrast to Cr(VI), which, as chromate (CrO_4_^2−^) or dichromate (Cr_2_O_7_^2−^), is toxic and competitive with anions such as sulfate and phosphate [38,56]. The molecular mechanisms of toxicity as well as potential essentiality of many other metals, such as Ni and V, and nonmetals, such as Si and boron (B), are still a subject of debate [57,58,59,60]. The first step in understanding trace element metabolism and their role in disease pathogenesis is to determine their distribution in different compartments and identify sites of accumulation, trace element deficiencies, or elemental imbalances. In recent years, much research has focused on predicting factors influencing metal speciation [61] and comparative metallomics, which involves monitoring changes in metallomics over time in healthy and diseased tissues from different populations suffering from various diseases [59,61,62,63,64,65,66,67,68,69,70,71,72,73,74,75,76]. Extensive metallomics studies allow us to understand the role of mineral imbalances in the pathogenesis of these diseases and dysfunctions, including neurodevelopmental disorders (NDDs) such as schizophrenia (SZ), attention deficit hyperactivity disorder (ADHD), autism spectrum disorder (ASD), epilepsy, and drug and alcohol addiction [77,78,79,80,81,82,83,84]. A review of metallomics studies reveals a major trend in contemporary research, which, in addition to identifying changes in tissue macro- and micronutrient levels, also seeks interelemental correlations. Toxic elements, including Cd, have been found to disrupt cellular metabolism of Ca, Fe, Mg, Zn, and Cu. Conversely, deficiencies of essential trace elements, such as Zn, Co, Cu, K, Fe, Mg, Mn, Se, and Zn, not only induce epigenetic changes but can also enhance the absorption of toxic metals [48,85].

## 3. Analysis of the Elemental Composition of Biological Samples

### 3.1. Sample Pretreatment

Biological fluids have a complex matrix rich in organic and inorganic compounds. In addition to their multicomponent matrix, they are characterized by very low concentrations of most trace elements (up to ng/L). The sample digestion step is crucial for subsequent chemical analysis using spectroscopic techniques. Sample preparation for trace element determination can be accomplished by acid digestion (nitric acid and hydrogen peroxide), alkaline digestion (tetramethylammonium hydroxide); ultrasonic matrix destruction; ultraviolet photolysis; protein denaturation (trichloroacetic acid or nitric acid); and microwave digestion. Ivanenko et al. [86] reviewed trace element analysis in biological fluids. In the case of elemental analysis of body fluids, i.e., urine, serum/plasma and whole blood, the sample may be diluted with an appropriate diluent and optionally with internal standards.

The direct analysis of samples without a digestion step is very attractive. Jones et al. [87] developed a sample preparation method for the determination of Pb, Cd, and Hg in whole blood using ICP-MS. The authors used sample dilutions in an alkaline diluent without acid digestion, additional sonication, or centrifugation. The optimized diluent composition (0.4% (*v*/*v*) tetramethylammonium hydroxide (TMAH), 1% ethanol, 0.01% ammonium pyrrolidinedithiocarbamate (APDC), 0.05% Triton X-100™, 5 μg L^−1^ Rh, Te, Ir) minimized memory effects from high-concentration samples. The sample volume was 50 μL of whole blood and the LOD reached the values: 0.07 μg dL^−1^ for Pb, 0.10 μg L^−1^ for Cd, 0.28 μg L^−1^ for Hg, 0.99 μg L^−1^ for Mn and 24.5 μg L^−1^ for Se. Navarro et al. [88] developed a method for preparing whole blood samples for the determination of Pb by graphite furnace atomic absorption spectrometry (GFAAS). Blood was diluted 10 times with 0.01% Triton X-100; and a mixture of 0.6% m/V NH_4_H_2_PO_4_ and 0.15% m/V Mg(NO_3_)_2_ in 0.01 M HNO_3_. The sample volume was 10 µL of blood and the LOD values were 1.2 µg L^−1^ of Pb (pyrolytic graphite tube) and 0.7 µg L^−1^ of Pb (L’vov platform).

Solid samples preparation for further elemental analysis requires mineralization, which is the decomposition of organic substances into simple inorganic compounds, which are formed through oxidation and evaporation. Elements such as H, C, S, and N are converted to gaseous forms, while other elements remain as oxides and salts. Mineralization can be performed in a closed or open system, wet or dry [89,90,91]. In most cases, acids or acid mixtures such as HCl, HNO_3_, H_2_SO_4_, HClO_4_, and HF, with or without H_2_O_2_, are used for digestion at elevated temperatures.

Historically, the oldest method of digestion is wet digestion in an open system. Currently, wet digestion in a closed system using microwaves is more popular due to its several advantages over other techniques, especially open digestion in a heating block. Microwave digestion utilizes chemical processes (acid decomposition), thermal energy (temperature), and microwave energy (2450 MHz electromagnetic radiation) for digestion.

Closed and open digestion systems differ significantly in terms of process support (closed: thermal conductivity, microwaves; open: additional UV, ultrasound), vessel type (closed: reusable: teflon, quartz, or glassy carbon, requiring cleaning with hot acid vapors and steam; open: disposable DigiTube vessels), and degree of digestion (closed: high; open: low). The closed system is characterized by a shorter digestion time (30–60 min) and the ability to handle sample sizes < 0.5 g dried weight, but is associated with higher costs and is not suitable for samples that generate a lot of gas. The open system, although slower (60 min) and subject to loss of gas products, is less expensive and allows for larger sample sizes (0.5–2.0 g). Dry digestion involves oxidation with supplied oxygen, which leads to the decomposition of organic matter into simple inorganic compounds. Dry combustion digestion involves oxidizing samples at high temperatures (450–550 °C) for 3–5 h, which ensures the decomposition of the organic matrix but can lead to elemental and contaminant losses; it requires subsequent acid digestion of the ash. Low-temperature oxygen plasma digestion, although time-consuming, allows for the determination of trace elements thanks to combustion below 150 °C. Oxygen bomb combustion and the Schöniger method are closed-system methods under pressure in which gaseous products are absorbed by aqueous solutions or by sulfuric acid or hydrogen peroxide, respectively. Dynamic combustion allows for the digestion of larger samples (up to 10 g) using a quartz apparatus, where gaseous products are absorbed and solids (ash) are digested in acids. Alloying at 500–1200 °C with fluxes produces a homogeneous product, but high salt content can cause interference. However, the method is expensive and carries a risk of contamination.

The vast majority of authors prefer sample digestion using microwave digestion in closed vessels, which occurs at high temperatures and pressures, which increases the reaction rate and eliminates the possibility of loss of trace elements that form volatile hydrides and/or halides, such as As, B, Cr, Hg, Sb, Sn, and Se. Furthermore, it uses smaller amounts of reagents, making it more economical.

### 3.2. Selected Analytical Techniques

Many analytical techniques are known for the quantitative determination of metals and selected nonmetals and metalloids. Due to limitations, the choice of technique should be tailored to the specific task and depends on the sample matrix, the level of the elements being determined in the sample (concentration range), and the type of elements being determined.

AAS became widespread in the 1960s. A hollow cathode lamp (HCL) is most often used as a source of monochromatic radiation with a characteristic wavelength typical of the element being determined. Therefore, it is necessary to have separate lamps for each element being determined. AAS instruments differ in the method of sample atomization. In flame atomic absorption spectrometry (FAAS), atomization occurs in a flame (air-acetylene, nitrous oxide-acetylene), while in electrothermal atomic absorption spectrometry (ETAAS), it occurs in a graphite furnace, where the sample is electrically heated in an inert gas (argon) that prevents oxidation of the graphite tube and purges the fumes from the tube. Chemical elements that form volatile hydrides, i.e., As, Bi, Cd, Hg, In, Pb, Sb, Se, Te and Tl are determined by hydride generation atomic absorption spectroscopy (HGAAS), while mercury (Hg) is usually determined using cold vapor atomic absorption spectrometry (CVAAS) [92]. The three versions of atomic absorption spectrometry—FAAS, GFAAS, and GHAAS/CVAAS—differ primarily in detection range, required sample volume, cost, and susceptibility to chemical and spectral interferences. FAAS, characterized by intermediate costs, is less sensitive (detection limits in the ppm range) and more affected by chemical and spectral interferences, but requires a larger volume of sample (2–3 mL) in a shorter time (10–15 s/element). GFAAS, on the other hand, achieves very good detection limits (ppb) and requires a small volume of sample (on the order of a few microliters). GFAAS, due to its better control ensured by longitudinal Zeeman background correction and the use of matrix modifiers, is more resistant to matrix background errors [93]. GFAAS has disadvantages, including longer analysis times due to more complex stages, such as drying and ashing, as well as higher instrument costs. GHAAS/CVAAS also offers very good detection limits (ppb-ppt) and minimal interferences, requires a large sample volume and is expensive, and the analysis time is the longest; however, it can be integrated with automated sample introduction (flow injection) for faster throughput and reduced sample/reagent consumption [94,95].

Inductively coupled plasma spectroscopy (ICP) is a group of analytical techniques that utilize a high-temperature plasma to excite or ionize a sample. Combining ICP with mass spectrometry (MS) is currently most commonly used in practice for the quantitative detection of multiple elements simultaneously in trace amounts in biological samples. Identification is based on the mass spectrum, which represents the intensity of the mass-to-charge ratio (*m*/*z*) of the cation formed in the plasma. ICP-MS provides rapid and sensitive analysis of many elements, including isotopes, with low detection limits in the parts-per-billion (ppb) or even parts-per-trillion (ppt) range and a wide linear range, enabling measurement over a wide range of metal concentrations in samples [96,97]. ICP-MS requires expensive equipment and appropriate sample preparation, as matrix effects can interfere with the results. ICP-MS is equipped with a special system for spraying liquid samples in a nebulizer-spray chamber and a system for laser evaporation of solid samples using a laser beam (LA-LCP-MS) [98]. Laser ablation (LA) coupled to ICP-MS (LA–ICP-MS) is used for in situ analysis and quantitative imaging of metal distribution in biological samples. LA-ICP-MS provides high spatial resolution and excellent sensitivity compared to sample solution analysis [99,100]. Coupling ICP with optical detection, i.e., inductively coupled plasma emission spectroscopy (ICP-OES), enables identification through spectrometric measurement of emitted light at wavelengths characteristic of individual elements. Another optical technique, rarely used in the analysis of biological samples, is laser-induced breakdown spectroscopy (LIBS). The basic theory behind LIBS is plasma emission spectroscopy, which describes the spectral emission of a plasma produced by a laser-induced breakdown.

Techniques based on synchrotron X-ray fluorescence (XRF), thanks to their high resolution, are increasingly used for element mapping in tissues, cellular compartments, and single cells [100,101]. XRF involves excitation of the sample with high-energy X-rays and measurement of the characteristic fluorescence. Advantages of this technique include high accuracy, precision, no need for special sample preparation, the ability to analyze many elements from Be to U, a detection limit at the level of ppm, no sample destruction, and the ability to analyze solid samples. The weaknesses of this technique include lower sensitivity for light elements, the inability to detect metals in trace amounts, and interference from other elements. Clavijo Jordan et al. investigated the distribution and transport of Zn at the organ level using microsynchrotron X-ray fluorescence (μSR-XRF) using a gadolinium-based contrast agent reactive to Zn(II) [102]. Another example of this technique is the mapping of Mn using nanosynchrotron X-ray fluorescence (nano-SXRF), which allowed for the acquisition of an image of the subcellular distribution of this redox-active metal. While XRF uses an X-ray beam for excitation [103], EDS (energy dispersive spectroscopy) relies on excitation by an electron beam for elemental analysis. A scanning electron microscope (SEM) equipped with EDS provides information on structure and morphology, but also allows for the assessment of the elemental composition of the sample. The electron gun generates a beam of primary electrons, which, upon interaction with the sample, emits secondary electrons, X-rays, and cathodoluminescence. An EDS detector is used to analyze the emitted X-ray radiation (energy, wavelength, and intensity of spectral lines) pointwise, along a designated line, or for surface elemental mapping. EDS can be coupled with both SEM and TEM. X-ray absorption spectroscopy (XAS) allows for the determination of oxidation states of metals in cells, the type and number of neighboring atoms, and the coordination motif of the probed metal [104]. Electron energy loss spectroscopy (EELS) is another research technique based on the interaction of a high-energy electron beam with the sample material. EELS can be integrated with Scanning Transmission Electron Microscopes (STEM) or Energy-Filtered Transmission Electron Microscopes (EFTEM) to generate elemental maps and spectrum images, but its potential is rarely fully utilized, especially in the case of biological specimens. Although most toxicology laboratories are equipped with AAS, GFAAS, HG (CV) AAS, ICP-MS, atomic fluorescence spectrometry (AFS) as a highly sensitive (LOD below the µg L^−1^), and selective technique (element-specific process) is also suitable in toxicological studies for analyzing trace amounts of toxic elements [104,105]. Especially, AFS is dedicated to speciation hydride forming elements (As, Se and Sb) and Hg [106]. AFS is suitable for detecting one element at a time, which slows down the process when the sample contains multiple elements of interest.

Particle-induced X-ray emission (PIXE) techniques use a proton source from the radioactive decay of Cm244 to excite and measure the characteristic X-ray radiation of the elements. PIXE offers high sensitivity (µg/g range), can analyze multiple elements simultaneously, and is often a non-destructive method, making it valuable for studies of biological samples. Chassot et al. examined metallic contamination in surrounding tissues with metallic elements released from joint prostheses [107]. Huszank et al. used particle-induced X-ray and gamma-ray emission spectrometry (PIXE, PIGE) techniques to determine trace and major elements in human blood samples (whole blood, serum, and plasma) [108]. In Neutron Activation Analysis (NAA) and Gamma Spectrometry, the sample is irradiated with a neutron beam from a nuclear reactor, and the gamma emission from the decay of radioactive isotopes is measured. This sensitive technique is used by the National Institute of Standards and Technology (NIST) for standard certification. NAA is used for the analysis of gold (Au) and silver (Ag) nanoparticles (NPs) by making them radioactive for detection. Both techniques are cumbersome to use and require radiological safety measures.

Imaging techniques for the uptake and subcellular distribution of metals in cells or organs are increasingly appreciated tools, allowing for a broader understanding of the mechanism of action of metal derivatives. Nanoscale secondary ion mass spectrometry (NanoSIMS) is an example of a technique designed for in situ elemental and isotopic analysis. NanoSIMS provides high resolution and sensitivity, which has been proven to track the transformation of metallo-medicines containing platinum (Pt) and ruthenium (Ru) [109,110]. Multiplexed in-cell imaging is provided by a combination of LA and mass cytometry, known as Imaging mass cytometry (IMC) [111]. IMC has been used to study the distribution of Pt in tissues after cis-Pt treatment [112].

**Figure 2:**
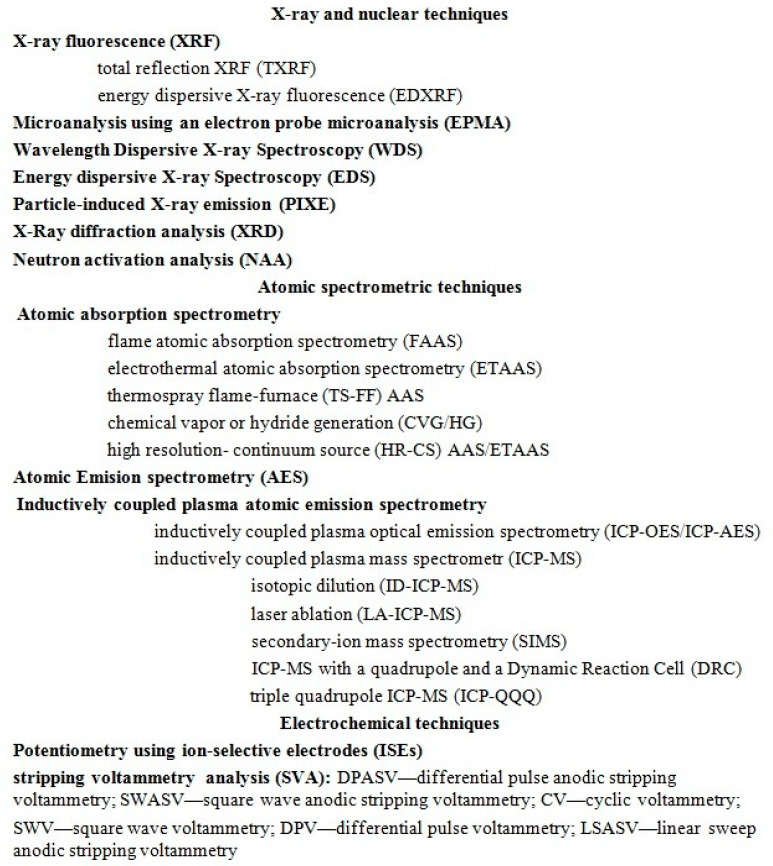
Analytical techniques for the determination of trace elements in biological samples.

Electrochemical techniques rely on electrochemical reactions between metal ions and selective electrodes. Electrochemical techniques have the disadvantage of being susceptible to matrix interference and requiring frequent electrode maintenance. A recent review by Shi et al. from 2025 details recent advances in electrode surface modification with metal nanomaterials (such as titanium dioxide (TiO_2_), copper oxide (CuO), ZIF-8, MXene, etc.) to improve sensor performance [113]. The sensitivity of electrochemical techniques, such as voltammetry, which detects heavy metal ions with a detection limit of 0.1 nM, and electrochemical sensors based on metal-organic frameworks (MOFs) achieves subnanomolar sensitivity in situ [114]. Among electrochemical techniques, anodic voltammetry (ASV) with a mercury electrode in the form of a hanging drop or thin film, also known as sequential injection-anodic stripping voltammetry (SIA-ASV), is noteworthy. Voltammetry is a sensitive, inexpensive, and useful technique for detecting trace amounts of metals. Its operating principle involves the accumulation of reduced ions in the form of an amalgam on a working electrode under the influence of an applied reduction potential. Upon application of an oxidation potential specific to the analyte, a cathodic current is generated proportional to the analyte concentration. However, ASV can only detect approximately 20 elements, whereas ICP-MS can detect over 70 [115]. The classification of analytical techniques used in the analysis of chemical elements in samples is presented in the diagram in Figure 2.

Chromatographic techniques, including ion chromatography (IC) such as cation exchange, anion exchange, and ion exclusion, as well as reversed-phase ion interaction chromatography (RP-IC), chelate chromatography, and multidimensional or multimodal chromatography (for example, combining ion exchange with ion exclusion or using two or more columns), are viable options for analysis. These methods can utilize conductometric or spectrometric detection, linking the detection process with separation [116]. Compared to analyses based on spectroscopic techniques, IC offers insight into metal speciation, allowing for the differentiation of metal oxidation states, for example: Cr(III)/Cr(VI), Fe(II)/Fe(III), or As(III)/As(V).

Lateral flow assay (LFA) technology, thanks to biosensor technology, has expanded its applications in the detection of heavy metal ions [117]. Metal detection utilizes key recognition mechanisms, such as DNA probes, nucleic acid enzymes, aptamers, and antigen-antibody binding. Applications of this technology so far include environmental monitoring and food safety.

## 4. Contamination of Tobacco Products with Heavy Metals

Tobacco contains over 7000 harmful substances. At least 69 of these chemicals are classified as carcinogens [118]. Cigarette smoke contains many toxic volatile organic substances, such as nitrosamines, polycyclic aromatic hydrocarbons, and toxic metals [2,118]. Fowles et al. [119] presented a list of approximately 158 carcinogenic or potentially carcinogenic chemicals present in cigarette smoke. The authors indicate that the cancer risk index (CRI) depends primarily on the contribution of 1,3-butadiene, acrylonitrile, As, and acetaldehyde. Among the toxic substances are also toxic metals that can cause inflammation, sensitization, and carcinogenesis. Metals such as As, Be, Cd, and Ni are group I carcinogens, as defined by the International Agency for Research on Cancer (IARC) [120]. Metals contained in tobacco products reach tobacco leaves through absorption from the soil [121]. Tobacco has been shown to accumulate heavy metals. Plants absorb metal ions and compounds from the soil through their roots, which are then transported to the leaves [122]. The efficiency of mineral uptake from the soil depends on the plant’s growing conditions, soluble organic matter, soil type and pH, fertilizers used, and the geographical location of cultivation [123,124]. Heavy metal contamination varies from country to country, depending on the location of cultivation and tobacco processing technology [125]. Therefore, the origin of tobacco may play a significant role in the level of cigarette contamination [126,127,128,129,130]. Heavy metal contamination of soil has become a global problem as the agricultural and industrial sectors are rapidly developing. Elements such as As, Cd, Cr, Hg, and Pb enter the soil through various routes, including sewage irrigation, atmospheric deposition, mining activities, and the use of chemical fertilizers [131].

Another source of tobacco contamination may be the manufacturing process and additives, such as flavorings or humectants [132]. Therefore, in March 2012, the U.S. Food and Drug Administration (FDA) published guidelines for reporting harmful ingredients in tobacco products, including the content of metals such as As, Cd, Cr, Ni, and Pb [133]. Tobacco products have been analyzed for trace toxic metals. Numerous analyses of heavy metals have been conducted in numerous cigarette brands worldwide [134,135,136]. A recent review of heavy metal studies in cigarette brands produced from 2000 to September 2024 provides average levels of toxic heavy metals (As, Cd, Cr, Hg, and Pb) and assesses associated health risks to consumers [137]. An example of such a study is another study performed using the ICP-MS technique on the content of toxic metals, i.e., As, Be, Cd, Cr, Co, Pb, Mn, Hg, and Ni in 50 commercial tobacco products available in the USA, described by Fresquez et al. [138]. The determined mean levels of Mn ranged from 131 to 245 μg/g; Cr from 1.4 to 3.2 μg/g, As from 0.22 to 0.36 μg/g, Cd from 1.0 to 1.7 μg/g, Ni from 2.1 to 3.9 μg/g and Pb from 0.6 to 1.2 μg/g. As reported by IARC, the detected metals have a carcinogenic effect [139]. The toxicity of these metals depends, among others, on volatility, which is why nickel and cobalt, for example, pose a lower risk as they are less volatile. In turn, even if Cd and Pb concentrations were lower in dried tobacco, due to the volatility of Cd compounds (chloride, oxide) or lead chloride, transport in smoke is more efficient [140]. Ni causes a number of different forms of cancer, especially in the respiratory tract. The main mechanism responsible for this activity is that Ni is mutagenic and induces sister chromatid exchange [141]. The amount of Ni varies significantly in cigarettes and can range from 0.078 mg to as much as 5 mg [142].

Tobacco is also characterized by high concentrations of Li, which exhibits a broad spectrum of neuroprotective effects, including enhanced autophagy and reduction of α-synuclein, as confirmed in preclinical models of Parkinson’s disease (PD) [143]. It is hypothesized that inhaled Li from tobacco smoke may be responsible for the lower incidence of PD and melanoma, and this mechanism may be mediated by inhibition of GSK-3β and activation of the β-catenin pathway. This hypothesis requires verification in clinical trials assessing the effect of Li therapy on the progression of PD and melanoma.

Tobacco radioactivity originates from the accumulation of natural radionuclides such as potassium-40 (^40^K), polonium-210 (^210^Po), lead-210 (^210^Pb), radium-226 (^226^Ra), thorium-232 (^232^Th), and uranium-238 (^238^U), primarily through absorption from soil and deposition on leaves. Phosphate fertilizers can increase their content in soil and tobacco plants [144]. During tobacco combustion, radioisotopes are transferred to smoke, which, when inhaled, is deposited in the bronchi and lungs of smokers, causing harmful internal exposure through alpha particle emission [145], contributing to an increased risk of lung cancer, heart disease, and other conditions. Studies by Karagueuzian et al. [146] and Laking [147] analyze the content of these radioisotopes in cigarettes, smoke, and human tissues. Karagueuzian et al. [146] published data on cigarette radioactivity resulting from the content of radioactive ^210^Po in cigarette smoke and assessed the long-term risk of lung cancer caused by alpha particle deposition in the lungs of heavy smokers. Laking [147] presented the results of studies on the content of ^210^Po and ^210^Pb in cigarettes, tobacco leaves, cigarette smoke, and human respiratory tissues. In a review by Felix and Ntarisa [148] from 2024, 40 publications were analyzed regarding the level of radioactivity in tobacco cigarette brands and the associated radiation effects on smokers. The studies most often used alpha particle and gamma radiation spectrometry to assess the activity of radioisotopes. The mean radioactivity included the isotopes ^210^Po, ^210^Pb, ^40^K, ^232^Th, ^232^U and ^226^Ra, with values of 20.4 (0.4–128.6), 15.4 (2.0–78.8), 630.8 (1.2–1330.0), 8.1 (0.3–41.0), 15.2 (0.2–82.0) and 5.9 (2.0–16.0) mBq/g, respectively. The annual effective doses, estimated assuming smoking 20 cigarettes per day, were 295, 74.1, 5.80, 889.7, 192.6, and 90.1 μSv/year, respectively, with ^232^Th radioisotopes contributing the largest amount to the annual effective dose and risk. The mean annual effective dose remains below the International Commission on Radiological Protection (ICRP) 103 reference level of 1000–20,000 μSv/year. The excess lifetime cancer risk (ELCR) and lifetime cancer risk (LCC) were also below the threshold values. However, the literature sources cited by the authors indicate that cigarette smoking remains the leading cause of lung cancer worldwide, contributing to up to 70–90% of lung cancer cases.

Cigarette smoke consists of particulate matter suspended in the gas phase. Over 3500 chemicals have been identified in these particulate matter, including tar, nicotine, polycyclic aromatic hydrocarbons, and heavy metals [149].

Matassa et al. [150] used microscopic imaging to provide direct evidence that metallic, inorganic microparticles are transported with the smoke stream to fibrous filters in the mouthpiece, forming nanostructures up to 150 µm containing compounds of V, Cr, Fe, Ni, Cu, U, Mn, and osmium (Os), among others. The authors point out that the porosity of the filters does not hinder the passage of toxic volatile substances. This research provides a basis for further work on creating microfibers capable of capturing toxic particles in the gas phase to protect human health.

The efficiency of transport of toxic metals from tobacco filler into cigarette smoke during smoking depends on many factors, but will always be proportional to their concentrations in tobacco [151,152]. Some components have been shown to penetrate smoke better than others. The highest rates of penetration from tobacco into cigarette smoke were found for Tl (85–92%) and Cd (81–90%), followed by Pb (46–60%) and As (33–44%). Studies show that the concentration of metals in cigarette smoke ranges from almost 1% (As) to as much as 22% (Cd) [123,153,154,155,156]. Therefore, it seems understandable that significantly higher levels of As, Cd, and Pb were found in the lung tissue of smokers compared to nonsmokers, demonstrating that smoking increases the levels of these metals in the lungs and may contribute to the carcinogenic effects of cigarette smoke [156]. Smoking plays only a minor role as a source of Hg emissions. Suzuki et al. [157] reported that only 5–7 ng Hg from one cigarette enters the smoke. The concentration of V in cigarettes averages 1.11 mg/cigarette, of which approximately 31.3% enters the smoke [158]. V is known to cause local irritation of the eyes and upper respiratory tract (such as rhinitis, cough, conjunctivitis, chest pain) and has insulin-like properties [159,160]. It is also suspected of causing neurobehavioral disorders that affect attention and visuospatial abilities [161].

Still, few studies have quantified the trace metals in secondhand smoke (SHS) and thirdhand smoke (THS) contributing to nonsmokers’ health risks. It is known that trace elements are released during smoking primarily as particulate matter. A recent study by Matt et al. [162] demonstrated a correlation between Pb and Cd concentrations and nicotine in settled dust from smokers’ homes, indicating that passive smoking may be a significant source of exposure to these elements. In a 2024 study by Tang et al. [163], the emission and deposition rates of 28 trace metals, including Cd, As, Cr, Mn, Be, and Se, were investigated indoors using inductively coupled plasma triple quadrupole mass spectrometry (ICP-QQQ-MS). The authors conclude that average indoor concentrations of Cd, As, and Cr, even more than 3 h after smoking cessation, exceed cancer risk thresholds or non-cancer reference exposure levels.

Studies of passive smokers indicate that they also accumulate metals emitted during smoking. Gatzke-Kopp et al. [164] demonstrated a correlation between cotinine and Pb and other metals in the saliva of children. Jung et al. [165] measured elevated levels of Cd and Pb in the blood of nonsmokers exposed to tobacco smoke. In a study by Drago et al. [166], air in the homes of smokers (41 homes) was examined and compared with air in control homes (32 homes). Smokers’ homes were found to have higher concentrations of cerium (Ce), lanthanum (La), Cd, and Tl in PM_2.5_. A study by Bohlandt et al. [167] presents measurements of Cd, Ce, and La concentrations in smokers’ homes and public places (pubs and restaurants). In smokers’ and control homes, 0.8 and 0.1 ng m^−3^ of Cd, 9.6 ng m^−3^ and 0.4 ng m^−3^ of Ce, 5.9 ng m^−3^ and 0.2 ng m^−3^ of La were measured, respectively; in public places, Cd concentrations ranged from 2.6 to 9.7 ng m^−3^, Ce concentrations ranged from 18.5 to 50 ng m^−3^, and La concentrations ranged from 10.6 to 23 ng m^−3^. The study by Na et al. [168] showed higher levels of rare earth elements (La, Ce) in the hair of non-smokers living with smokers.

Inorganic particles found in tobacco products were analyzed as a potential source of exposure due to smoking. SEM-EDS demonstrated the presence of aluminum silicate, phytolithic silica, and calcium compounds such as oxalates in tobacco products [169]. Mineral components in tobacco are transported in the smoke stream and, when inhaled, cause chronic inflammatory diseases of the oral cavity and lungs. It has been suggested that Cd from cigarettes is mostly associated with smaller airborne particles with an aerodynamic diameter below 1.8 μm [170]. Piadé et al. believe that approximately half of the Cd released during smoking is released in the form of organocadmium compounds, such as Cd(CH_3_)_2_, which could explain its volatility [171]. In the case of As content, while inorganic As(V) compounds predominate in tobacco leaves, both As(V) and As(III) are present in the main smoke condensate, indicating As reduction during combustion [172]. Studies on the analysis of tobacco of various origins to quantify the concentrations of toxic substances transported into cigarette smoke and inhaled by smokers are included in Table 1.

Cd is a metal frequently detected in tobacco products. It is a carcinogen associated with the etiology of chronic obstructive pulmonary disease (COPD) and nephrotoxicity. Suwazono et al. investigated the biological half-life of Cd in urine based on a 24-year study [180]. The estimated half-life was 13.6 years (9.0–28.2 years) in men and 13.9 years (9.6–25.6 years) in women. The creatinine-corrected values were 14.2 years (11.2–19.4 years) and 23.5 years (17.7–35.0 years), respectively. It should be remembered that Cd can accumulate in various tissues but is stored primarily in the liver and kidneys. The biological half-life of Cd in body tissues varies and can range from 10 to 30 years [180,181,182]. Cr, present in tobacco smoke, has toxicity dependent on its oxidation state. While Cr (VI) is an IARC group 1 carcinogen [183], Cr (III) is only an allergen [184]. Pb is an IARC group 2A carcinogen [185]. Pb is a known neurotoxic agent [186]. Mn is neurotoxic [187]. Co is an IARC group 2B carcinogen. Metals such as Mn and Se, Fe, and Cu are proinflammatory and allergenic in excess [188,189,190]. Ba and its oxide are non-volatile, so despite their presence in tobacco, they are not transported into smoke [173]. However, other authors suggest that Ba may enter the human body via inhalation exposure vectors. Ba may be a component of particulate matter, and similarly to oral ingestion, the rate of absorption through respiratory membranes depends on the chemical form [191].

Al in the form of soluble compounds is readily bioavailable to both plants [192] and humans [193]. Exposure to Al compounds through absorption, inhalation, or ingestion is neurotoxic, as long-lived postmitotic cells, such as neurons, are particularly susceptible to Al accumulation [193]. It should be noted that Al uptake from the gastrointestinal tract is quite low and does not exceed 0.01%, however, over time Al accumulates in the brain, bones, and other tissues [194]. One route of human exposure to Al is through inhalation of tobacco smoke, either during active or passive smoking [195]. Although Al compounds are mostly non-volatile, combustion of Al-containing tobacco causes transport of insoluble aluminum silicates with the smoke stream as particles or gaseous complexes to the oral cavity and lungs, where they accumulate and are engulfed by phagocytic cells such as bronchoalveolar and interstitial macrophages [196,197,198,199,200].

## 5. Major Health Risks Associated with Exposure to Tobacco Smoke

Tobacco-related diseases include incurable respiratory diseases, such as COPD and comorbidities associated with COPD, such as chronic bronchitis, emphysema, and recurrent bacterial lung infections. Currently, the WHO predicts that by 2030, COPD will become the third leading cause of disease-related deaths worldwide [7]. Furthermore, smoking is associated with a causal relationship with an increase in the incidence of malignant tumors, including lung cancer, laryngeal cancer, oropharyngeal cancer, cardiovascular diseases, including coronary heart disease, as well as type 2 diabetes and reproductive function [1,2,3,4,5,6,7,151,201,202,203,204]. Metals in cigarette smoke play a significant role in the process leading to damage to the vascular endothelium, which is a major factor in the development of atherosclerosis, which is responsible for the dramatically increasing number of deaths.

### 5.1. Addiction to Smoking Cigarettes

**Figure 3:**
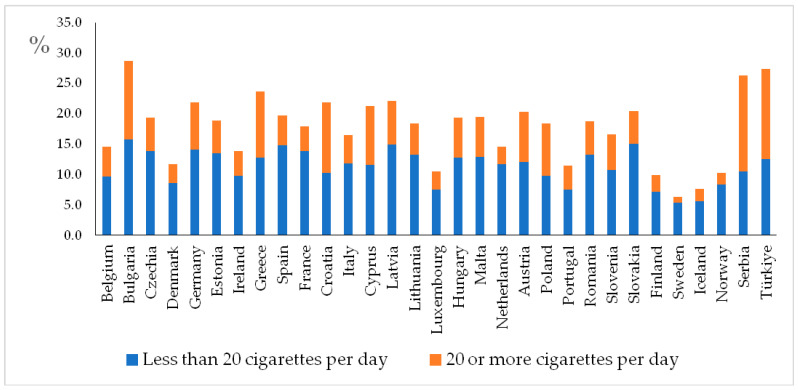
Share of cigarette smokers among the adult population over 15 years of age, by level of consumption in 2019 [214].

Nicotine is known to be a highly addictive neurotoxin [205]. The lethal dose (LD_50_) of nicotine is approximately 1–1.5 mg per kg of body weight. The median LD_50_ for nicotine in adults is assumed to be 0.8 mg/kg [206]. The Royal Children’s Hospital in Melbourne, in its clinical practice guidelines, states that the potentially lethal dose of nicotine for children is 0.5 mg/kg [207]. Difficulty quitting smoking stems from the need for self-medication, providing a temporary mood boost and stress relief. Because nicotine can reach the brain in seconds, this effect is almost immediate. Smoking provides relaxation and improves mood through the release of dopamine. On the other hand, smoking increases adverse stress symptoms, such as heart rate and blood pressure, which result from nicotine’s effect on the hypothalamic-pituitary-adrenal (HPA) axis and increased cortisol levels. Quitting smoking is associated with nicotine withdrawal stress, which can be alleviated by antidepressants [208,209]. Understanding the mechanisms of nicotine addiction has involved proteomic analyses, which assessed the effect of nicotine on specific genes or proteins [210,211]. Proteomic studies of smokers’ saliva identified protein markers, among which the levels of stress-related proteins, fibrinogen alpha, cystatin A, and sAA, were twice as high in smokers compared to nonsmokers [212]. A number of proteins (AMY1, CAH1, CAH6, BPIA2, BPIB1, TCO1, NGAL, GSTO1 A2ML1, TGM3, and SAP) have been found to be altered by smoking. In its ninth WHO report in 2023, tobacco smoking was declared a major public health threat, with nearly 8 million premature deaths annually attributed to tobacco use [4]. Data recently released in November 2023 by the Office on Smoking and Health, National Center for Chronic Disease Prevention and Health Promotion (NCCH), indicate that 28.3 million American adults smoke cigarettes, 16 million suffer from smoking-related diseases, and nearly half a million die annually from smoking. Healthcare expenditures for smoking-related diseases amount to $225 billion annually [213]. Eurostat data for 2019 regarding the share of cigarette smokers among people over 15 years of age is presented in Figure 3. As can be seen, 5.9% of the EU population aged 15 and older smoked at least 20 cigarettes a day, and 12.6% smoked fewer than 20. Among the EU Member States, only in Croatia did heavy smokers constitute the majority of daily smokers; heavy smokers also constituted the majority in Turkey and Serbia. By comparison, in the Netherlands and Sweden, less than one in five daily smokers was a heavy smoker. The planned data update will take place in July 2027.

The prevalence of cigarette smoking was even studied among university athletes at Thammasat University in Thailand, with a mean age of 19.8 ± 1.3 years [215]. The study confirmed that the percentage of smokers in the study population was relatively high. Smokers were predominantly male (70.6% vs. 29.4%, *p* < 0.001), who had higher levels of the measured indicator, exhaled CO (3.75 ± 3.08 ppm vs. 2.18 ± 0.73 ppm, *p* < 0.001). The division into the study and control groups was based on the results of the Fagerström test, a questionnaire used to assess nicotine dependence. According to the report of the National Health Fund in Poland on tobacco-related diseases from 7 July 2021, 8.5 million people smoked tobacco, which constituted almost 25% of people over 10 years of age [216]. The report indicates that in 2019, the value of health services allocated to the treatment of tobacco-related diseases increased by 47.4% compared to 2013.

### 5.2. Accumulation of Metals in Tissues by Tobacco Smokers

Tobacco smoke is known to contain numerous compounds, many of which are oxidants and pro-oxidants, capable of generating free radicals and exacerbating oxidative stress in vivo. There are intrinsic mechanisms for neutralizing free radicals, including enzymatic and non-enzymatic reactions. Copper-zinc superoxide dismutase (Cu-Zn SOD), glutathione peroxidase (GSH-Px), and catalase (CAT) are important components of the antioxidant enzyme system. It has been shown that tobacco smoking can alter the activities of these enzymes [217,218]. Because these enzymes contain metals as cofactors (the cytoplasmic Cu-Zn SOD enzyme contains Cu and Zn, the GSH-Px enzyme contains Se, and the CAT enzyme contains Fe), trace element homeostasis in smokers may be impaired by heavy metal intoxication [219,220]. It has been confirmed that chronic smoking leads to the accumulation of metals in tissues and body fluids, leading to health deterioration [156,164,165,197,219,220,221,222,223,224,225,226,227], although the number of studies on heavy metal intoxication resulting from smoking is still insufficient.

Tobacco smoke is a source of exposure to heavy metals, including Cd and Pb, which have a very long half-life in the human body [198,199]. The toxicity of metals detected in cigarette smoke is not limited to carcinogenicity; As and Cd are toxic to the cardiovascular system and kidneys, while Pb is toxic to the nervous system [228].

Gatzke-Kopp et al. in 2023 [164] examined the effects of exposure to passive smoking in children. Salivary cotinine levels in children aged approximately 90 months were selected as biomarkers of exposure using an immunoanalyzer and trace metal concentrations, i.e., Cr, Cu, Pb, Mn, Ni, and Zn, determined by ICP-OES. Interestingly, when the salivary cotinine concentration in children was higher than 1 ng mL^−1^, higher levels of Zn (95% CI: 0.183–0.619; *p* = 0.0003), Cu (95% CI: 0.206–1.104; *p* = 0.004), and Pb (95% CI: 0.424 to 2.459; *p* = 0.006) were observed compared to children whose salivary cotinine levels were lower than 1 ng mL^−1^. The study suggests that children exposed to tobacco smoke are at increased risk of heavy metal accumulation [164]. Rzymski et al. [222] were the first to examine eight metals (Al, Cd, Cr, Cu, Mn, Ni, Pb, and Zn) in aborted embryos. In samples collected from former smokers, significant increases (*p* < 0.05) were observed in the concentrations of Al (over 5-fold), Cd (over 2-fold), and Pb (over 2-fold).

Stojanović et al. [223] demonstrated elevated Ni levels in the blood and urine of smokers. Richter et al. [224] determined metal levels in urine. They found that smokers had significantly higher levels of Cd, Pb, Sb, and Ba compared to nonsmokers. Another study found that the mean content of Al, As, Cd, Hg, Ni, and Pb was significantly higher in the scalp hair and blood samples of diabetic patients compared to healthy controls, but the difference became statistically significant in smokers (*p* < 0.001) [178].

Tobacco may contain Tl, which is more toxic than Hg, Cd, and Pb, which are mainly absorbed from the soil and accumulate in leaves. The maximum dose of Tl should not exceed 15 µg, and the LD_50_ for humans is 8–12 mgTl × kg^−1^. Tl rapidly enters the bloodstream and is transported throughout the body, preferentially accumulating in nails, bones, and hair, but can also accumulate in the kidneys, liver, and brain. Tl is found in the tissues of smokers due to its presence in cigarette smoke, leading to higher levels in smokers’ urine and hair compared to non-smokers [225].

Biomonitoring studies show that smokers have significantly higher levels of Cd and Pb [226], but bioaccumulation of the metals has also been demonstrated in individuals chronically exposed to tobacco smoke pollution (also known as secondhand smoke or passive smoking) [227]. Cd exposure and accumulation have been associated with lung diseases as early as 1950 [229,230]. The National Health and Nutrition Examination Survey (NHANES) has been conducting continuous biomonitoring of Cd concentrations in urine since 1988 [224]. One of the original goals of the study was to estimate trends in Cd exposure between 1988 and 2008, influenced by population changes, particularly smoking. The report authors hypothesized that the observed decreasing trend coincided with decreasing rates of tobacco smoking.

However, the traditional focus on total body Cd burden, estimated through urine analysis, may not fully reflect the local Cd burden. Therefore, assessing local Cd accumulation in the lungs seems more relevant, considering tissue-specific Cd retention.

According to the WHO, Cd is a serious public health concern because it is a toxic heavy metal that increases the risk of kidney disease, osteoporosis, cardiovascular diseases, and various cancers, including lung cancer [231,232,233,234]. This metal is naturally accumulated in the tobacco plant (*Nicotiana tabacum*) [131,134]. This “hyperaccumulation” leads to very high Cd concentrations in tobacco leaves, relatively independent of soil content. Generally, the Cd content in tobacco leaves ranges from 1 to 2 μg g^−1^ dry weight, which translates to 0.5–1 μg Cd per cigarette. Cadmium oxide, produced during tobacco smoking, likely either accumulates locally in lung tissue or is absorbed into the systemic circulation, or both [231]. Absorption following inhalation into the lungs is thought to be significantly higher than absorption from food or the intestines, resulting in blood Cd concentrations that may be as much as four or five times higher and kidney concentrations as much as two or three times higher in smokers compared to nonsmokers [235,236,237]. However, very little is known about its local deposition in the lungs. Exposure to Cd in tobacco smoke has been associated with the development of numerous lung diseases [238], e.g., pulmonary fibrosis and emphysema, impaired ventilatory function, reduced gas exchange, and severe COPD in smokers. Cd accumulation poses a threat not only to the lungs but also to other organs, as this metal disrupts intracellular signaling, leading to impaired defense functions. For this reason, smokers are more susceptible to bacterial infections and chronic inflammation. However, the mechanisms of Cd’s pathogenic effects are poorly understood. Furthermore, it is uncertain whether the increased urinary Cd levels in smokers [239] and the observed increases in blood [240] and lung levels [239,241] are related to Cd accumulation in the lungs. It is difficult to distinguish between Cd-induced effects, the effects of other toxins present in tobacco smoke, or Cd-mediated effects. It is believed that chronic Cd accumulation in smokers’ lungs predisposes them to various lung diseases, especially since the half-life of Cd is very long and, according to various sources, can be even over 25 years [242,243]. ICP-MS elemental mapping of the bronchi and lungs of a group of smokers, compared to a control group, was published by a group led by J. Flieger [244]. The presence of Cd and Pb was noted in bronchial and lung samples, consistent with the predominant route of exposure: inhalation.

#### 5.2.1. Bronchi and Lungs

Studies on the determination of chemical elements in tissues collected from the bronchi and lungs of tobacco smokers are summarized in Table 2.

Local accumulation of Cd in the respiratory tract appears to be a critical component of the predisposition to lung disease among long-term smokers [238,249,250,251,252]. Kjell Torén et al. [251] showed that low-dose exposure to Cd was associated with an increased incidence of emphysema and impaired lung function in a group of 50–64-year-old residents of Gothenburg (n = 1111). The study population consisted of 45.3% (n = 336) never smokers and 17.4% current smokers (n = 129), with a mean of 16.7 pack-years for former smokers and a mean of 20.5 pack-years for current smokers. Never-smokers had significantly lower B-Cd concentrations (mean 0.22 µg L^−1^, median 0.19 µgL^−1^) compared to smokers (mean 1.02 µgL^−1^, median 0.85 µgL^−1^). The correlation (rs) between B-Cd and pack-years was 0.48 (*p* < 0.0001); among individuals with emphysema, it was 0.42 (*p* < 0.001); and among individuals without emphysema, it was 0.37 (*p* < 0.001). Cd accumulation is particularly important given that the biological half-life of Cd in the human body is up to 25 years, suggesting the possibility of significant Cd retention in the lungs of long-term smokers [253].

Studies by Flieger et al. [244] revealed elevated Al concentrations in the bronchi and lungs of smokers. Although Al compounds are mostly non-volatile, it is known that burning tobacco containing Al causes the transport of insoluble aluminum silicates into the lungs via the smoke stream as particles or gaseous complexes. Conversely, the concentrations of elements such as Cu, Rb, and Se were lower compared to control tissue. Additionally, an accumulation of rare earth elements (including Ce, praseodymium (Pr), neodymium (Nd), samarium (Sm), europium (Eu), dysprosium (Dy), and erbium (Er)) was observed in the lungs of smokers, which showed significant inter-elemental correlations with Al. It is known that Al is an element with no physiological function and is toxic even at low concentrations [193,254]. Se and Cu, in turn, are essential trace elements involved in the body’s antioxidant defenses as components of selenoproteins (GPx1, GPx2, GPx4), as well as selenium-dependent iodothyronine deiodinase enzymes (Dio1, Dio2) [255,256,257,258,259,260,261].

Cu deficiency weakens the body’s immune and antioxidant defenses, negatively impacts skeletal development, melanin synthesis, Fe metabolism, neurotransmission, and erythropoiesis [259,260,261]. Dyshomeostasis of many chemical elements is observed in states of oxidative stress and pathological conditions associated with inflammation [262,263,264,265,266,267,268].

Cr was detected in the lungs of smokers at the end of the 20th century [250]. The study was performed on a group of 45 Finnish individuals using plasma emission spectrometry (DCP-AES). Mean Cd and Cr concentrations were determined in nonsmokers, smokers, and former smokers. Cd concentrations were 0.4 (±0.4), 3.0 (±2.2), and 1.1 (±1.0) mg/g dry weight, but Cr concentrations were significantly higher at 1.3 (±0.9), 4.3 (±3.3), and 4.8 (±4.0) mg/g dry weight, respectively. In smokers, Cr concentrations increase with age and duration of smoking. It is important to remember that Cr can occur in the natural environment in the following forms: trivalent, potentially safe, and hexavalent (VI), highly toxic and carcinogenic. Studies indicate that Cr(VI) exposure is associated with an increased risk of lung cancer [183,184]. Other effects of Cr accumulation in the respiratory tract include ulcers, chronic rhinopharyngitis, impaired lung function, and emphysema (ICDA, Paris, 1997).

The role of rare earth elements in the body is still being studied. They are considered low-toxicity elements that can act as Ca analogs in biological systems [269,270,271]. Some of them, e.g., gadolinium (Gd), are used in diagnostics as a contrast agent in magnetic resonance imaging (MRI) [272,273].

#### 5.2.2. Liver, Kidneys, Prostate

In 2022, studies by Chen et al. published confirming that there is a high probability of developing non-alcoholic fatty liver disease associated with smoking [274]. Smoking is a risk factor for fibrosis and the development of hepatocellular carcinoma [275]. According to studies by Flieger et al. [244], smoker’s liver tissue contains significantly more toxic Pb and Tl than controls and less Cu. Pb, as one of the most toxic heavy metals [276], accumulates in the liver [277,278] and is responsible for its damage by generating oxidative stress [279]. The effects of Tl on the human body have not been described in sufficient detail, yet the toxicity of this element is one hundred times higher than that of Hg. The Tl^+^ ion not only forms complexes with S-containing ligands but also replaces K in enzymes and protoplasm (the ionic radii and properties of K^+^ and Tl^+^ are similar), shifting the Na^+^/K^+^ balance and disrupting membrane equilibrium [280].

The levels and ratios of Se and Cd in the prostate, liver, and kidneys of nonsmokers and smokers were studied by Schöpfer et al. [281] based on analysis of post-mortem samples from 129 men aged 15–99 years, collected from three different organs (prostate, liver, kidneys), and urine using AAS with the graphite furnace technique and longitudinal AC Zeeman effect background correction. In heavy smokers, prostates contain more Cd than prostates of nonsmokers, which occurs in stoichiometric excess relative to Se. The authors suggest that Cd accumulation in the prostate of smokers may contribute to the development of prostate cancer. In organs such as the kidney and liver, Cd levels also tend to exceed Se levels, but in these organs, Cd is detoxified by sequestration by metallothioneins (MTs). The mean Cd concentration of 240 µmol/kg in the kidneys of smokers was almost four times higher than in nonsmokers, ranging from 11 to 750 µmol/kg. The mean Se content was 10.4 µmol/kg, twice as high as in nonsmokers, ranging from 2.7 to 10.5 µmol/kg. Other studies also point to the fact that, through interaction with Cd, Se exerts a protective effect against prostate cancer [282]. Drasch, Schöpfer, and Schrauzer observed that the Se/Cd ratio declines more steeply with age in smokers compared to nonsmokers. In the prostates of some smokers, Se/Cd ratios even reached values of <1, indicating a stoichiometric excess of Cd over Se [282].

Studies in mice have shown that Ca can also mitigate Cd toxicity to the liver and kidneys [283]. The most beneficial protective effect was demonstrated by a concentration of 100 g/kg of dietary Ca. Cu supplementation may also have a protective effect against Cd-induced oxidative stress in the liver, kidney, and placental tissues of pregnant rats and their fetuses [284]. Cu supplementation also prevented Cd-induced decreases in reduced glutathione (GSH) levels and SOD, and CAT activity.

#### 5.2.3. Brain

Using AFM and NMR imaging, fluorescence, and mass spectrometry, Wallin et al. [285] investigated the effects of various chemicals associated with cigarette smoking (nicotine, polycyclic aromatic hydrocarbons, Cd, Cr, Pb) on Aβ aggregation in vitro. They found that both aromatic hydrocarbons and metal ions modulated the Aβ aggregation process through electrostatic interactions (Cd(II), Cr(III), and Pb(II)) or specific, transient coordination of binding to the N-terminal segment of Aβ (Pb(IV)). These results support the higher incidence of Alzheimer’s disease (AD) among smokers and indicate Pb as a possible environmental risk factor for the development of this disease. Pb is a neurotoxic element that accumulates in the tissues of smokers. Taiwo and colleagues [286] used a mouse model to investigate how neurotoxic metals from e-cigarette aerosol accumulate in the CNS at biologically significant levels. The study population of mice was exposed to a dose of e-cigarette aerosol comparable to human exposure or to a dose five times higher. After a two-month study, the olfactory bulb, anterior and posterior frontal cortex, striatum, ventral midbrain, cerebellum, brainstem, remaining brain tissue, and spinal cord were collected for analysis and metals were determined using ICP-MS. Even low-dose exposure resulted in increases in Cr, Cu, Fe, Mn, and Pb, measured in the striatum. Concomitantly, decreases in some essential metals were observed in the CNS, leading to endogenous metal dyshomeostasis. Therefore, long-term neurotoxic and neurodegenerative risks for e-cigarette users and bystanders are certain. Exposure to e-cigarette aerosol for over two months induces the accumulation of neurotoxic metals and alteration of essential metals in the mouse brain [287].

According to a study by Flieger et al. [244], statistically significant elevated levels of toxic metals such as Cd, Al, Cr, and Pb are observed in the brains of smokers, as well as changes in the content of elements involved in the body’s antioxidant defenses (e.g., Zn, Cu, Se), which may influence neurobiological processes. Examination of the elemental composition of various anatomical regions shows that statistically significant differences between the smoker and non-smoker groups were observed for the insula, which accumulates significantly more Al, and the nucleus accumbens septi and precentral gyrus, which contained significantly more Cd.

#### 5.2.4. Body Fluids

Levels of toxic metals such as Cd in body fluids (e.g., urine and blood) can be useful as biomarkers of exposure and potential harm. Many reports have shown a correlation between urinary Cd concentrations and smoking status, taking into account the number of packs smoked and the period of exposure [224,288,289,290,291,292,293,294,295]. Urinary Cd levels are clearly correlated with chronic exposure, while Cd concentrations in blood or serum indicate recent exposure and the number of cigarettes smoked per day [252,288,291,296,297,298,299,300]. The Rayyan study [299] examined Cd accumulation in acute (blood, B-Cd) and chronic (hair, H-Cd) storage sites in a group of 200 individuals, including 90 nonsmokers and 110 smokers, aged 18 to 65 years. The mean concentrations of H-Cd and B-Cd measured by ICP-AES were 0.417 µg/g and 0.292 µg/L, respectively. It was noted that the concentration of Cd was significantly higher in men than in women *(p* < 0.05). A significant correlation was found between smoking duration and H-Cd and B-Cd levels (r = 0.289, *p* < 0.05) (r = 0.303, *p* < 0.05). In the smoker group, a threefold increase in H-Cd and B-Cd levels was observed over a period longer than 10 years compared to the non-smoker group. There is evidence of an association between blood lead and cadmium levels with smoking-related changes in DNA methylation (DNAm) and an increased risk of mortality. Furthermore, the analysis showed an additive effect of these metals on smoking-related DNAm, as well as a synergistic effect on all-cause mortality [300]. According to the German Environmental Study [288], Cd concentrations in the blood of smokers were 3 to 6 times higher than in non-smokers. Torres-Sánchez et al. [301] examined blood Cd levels (BCd μg/L) in a group of approximately 700 men over 40 years of age from Mexico City using AAS. They found that the BCd level was dependent, among others, on current smoking status (β ≤ 8.5 packs/year vs. non-smoker = 0.46; 95% CI: 0.28–0.64 μg/L; *p* < 0.01) and high smoking status (β > 8.5 packs/year vs. non-smoker = 0.71; 95% CI: 0.56–0.87 μg/L; *p* < 0.01). According to a study by Adams et al. [291], smoking for 5 days increases Cd levels in blood, but not in urine. At the same time, quitting smoking causes a decrease in Cd concentrations in blood and urine, with the decrease being greater in men than in women, reaching 40% and 25%, respectively, within a year. Similar observations were reported by Wróblewski et al. [296] and Mérida-Ortega et al. [302].

In cigarette smokers, an inverse relationship between serum Cd and Zn levels was observed [296,303]. It appears that it is not the individual levels of these elements, but the Cd/Zn ratio that is indicative of an increased risk of disease. Fresquez et al. measured Zn concentrations to be insignificant compared to Cd in cigarette smoke particles [304]. According to Rodgman and Perfetti [123], 0.4–2.7% of Zn and 7–22% of Cd are transferred from tobacco to smoke during combustion. A preliminary study by Kim et al. [305] suggests that Cd absorption and accumulation may be dependent on Zn intake. Physiological levels of these elements in blood are dependent on gender and age [306,307]. Numerous studies have shown that Zn concentrations in blood, serum, and tissues are lower in tobacco smokers compared to nonsmokers [308,309,310,311]. In the study by Uz et al. [309], smokers had a mean serum Zn concentration of 14,150 µg/L compared to 16,050 µg/L in control subjects. Northrop-Clewes et al. [311] analyzed the results of studies on micronutrients (vitamins A, E, and C; the carotenoids; some of the B-vitamin group; and the trace elements Se, Zn, Cu, and Fe) in smokers and non-smokers. According to their findings, serum Se concentrations and erythrocyte glutathione peroxidase (GPx) activity were lower in smokers. In this review, the authors examined the impact of smoking on iron (Fe) status by analyzing several biomarkers, including serum iron, ferritin, hemoglobin, and a marker of iron-related oxidative damage: oxidized plasma low-density lipoprotein (LDL). Based on the collected data, they concluded that iron homeostasis in smokers seems to be disrupted, which is indicated by elevated hemoglobin concentrations. Moreover, CuZn-SOD) activity in erythrocytes and serum ceruloplasmin concentrations were higher in smokers compared to non-smokers. The authors suggest that this increase may be linked to the body’s anti-inflammatory response to smoking. Furthermore, serum Zn concentration was found to be decreased only in heavy smokers.

Kulikowska-Karpińska et al. described decreased urinary Cu concentrations in smokers [312]. Jasińska-Starczewska et al., in turn, observed higher plasma Cu concentrations in smokers compared to nonsmokers (122.5 ± 19.15 vs. 101.5 ± 16.2 μg/dL, *p* < 0.01) [313]. In the study by Choi et al. [314], the association between tobacco smoking and, among others, sodium (Na) intake was examined in a population of 6340 Korean men with a smoking prevalence of 44.1% and a mean number of pack-years of smoking of 13.2 (0–180). Rates of excessive Na intake were higher in the group exposed to smoking and alcohol drinking than in the group who never smoked or drank. The effect of smoking on electrolyte balance, i.e., Na and K, is not widely documented in research. In the study by Bushra et al. [315], blood samples from smokers and non-smokers aged 18–50 years were analyzed using the Atellica CH analyzer. The results showed electrolyte imbalance in smokers. Smokers, depending on smoking intensity, had elevated Na levels and decreased K levels, while Li levels were <0.10 mEq/L in all participants, regardless of smoking status. The measured serum Na concentration (men: 143.56; women: 144.4) mEq/L and serum K concentration (men: 3.82; women: 3.91) mEq/L were different compared to non-smokers (Na: men: 140.35, women: 140.75; K: men: 4.32, women: 4.23) mEq/L. These results are consistent with the study by Eliasson et al. documenting serum hypernatremia in chronic smokers [316]. Studies in animal models suggest that vasopressin secretion induced by chronic smoking causes renal water retention and increases Na concentration [317]. However, these differences in Na and K levels in smoker/non-smoker groups are not confirmed by epidemiological studies [318]. Smoking-induced hypokalemia causes the release of catecholamines, which activate Na^+^/K^+^-ATPase, which lowers serum K levels by transporting it into the cell [319]. Furthermore, activation of the renin-angiotensin-aldosterone system (RAAS) by nicotine increases urinary K excretion. Studies confirm the influence of comorbidities on the development of renal electrolyte imbalance in smokers. In medical emergencies, such as myocardial infarction, hypokalemia in smokers may not be confirmed [320] or may be exacerbated by treatment of hypertension with diuretics, which also cause K loss [321,322].

In the case of Li, Bushra et al. [315] suggested that smoking does not affect the dynamics of this electrolyte. Enderle et al. [323] also reported that plasma Li concentrations are approximately 1000 times lower than therapeutic levels even in populations of heavy smokers. This sheds new light on the assumption that smokers are at reduced risk of developing PD and melanoma due to inhalation of trace Li from tobacco [143,324]. The authors of the study [315] emphasize that differences in electrolyte levels between women and men should be taken into account when interpreting the results due to differences in electrolyte distribution, which are not eliminated by smoking. Few studies have reported measurements of metals in the saliva of adult smokers [325,326]. Environmental tobacco smoke may also be a source of increased exposure to heavy metals in children. Gatzke-Kopp et al. [164] demonstrated that children exposed to passive smoking had significantly higher levels of Zn (*p* = 0.0003), Cu (*p* = 0.004), and Pb (*p* = 0.006) in saliva in comparison to the reference group. The study authors warn that exposure to the highly neurotoxic Pb may contribute to learning and behavioral disorders, including attention deficit disorder.

#### 5.2.5. Skin, Hair

Human hair reflects the body’s mineral composition. This means that the concentration of elements in hair correlates with their internal levels in the body. Furthermore, the level of elements in hair is significantly higher compared to body fluids (blood, urine), which improves the quality of detection. Advantages of hair analysis include non-invasive sampling, ease of transport, storage, and purification [327,328,329,330,331,332].

A study by Afridi et al. [178] showed that mean values of Al, As, Cd, Hg, Ni, and Pb were two to three times higher in scalp hair and blood samples from smokers compared to nonsmokers (*p* < 0.001). It was also noted that smoking may interact synergistically with risk factors associated with diabetes. The samples were obtained from a population living in Dublin, Ireland, and analyses were performed using an inductively coupled plasma emission spectrophotometer.

Muchacka et al. [327] examined hair and nails collected from smoking and non-smoking women from the Małopolska region, aged 18 to 70. The samples were assayed for heavy metals: Cd, Pb, Ni, Mg, Zn, Fe, and Cu using the FAAS method. The study showed that more Cd, Zn, and Cu accumulated in hair than in nails in both groups. Smoking affects the accumulation of metals in hair and nails in the case of Mg, Ni, Cd, Fe, and Pb. Similar studies by Afridi et al. [328] determined Zn, Se, Cd, and Hg in scalp hair and blood from smokers and non-smokers with concomitant hypertension. The analysis revealed significantly increased mean concentrations of Cd and Hg in hair and blood samples from patients with hypertension compared to a control group of healthy individuals. Concomitantly, reduced levels of Zn and Se were observed in patients with hypertension, with a statistically significant difference observed in smokers (*p* < 0.001). Smokers, even without concomitant hypertension, demonstrated 2–3 times higher concentrations of the studied trace elements (TE) in hair and blood compared to nonsmoking controls. This suggests that exposure to trace elements through cigarette smoking may have a synergistic effect with other risk factors for developing hypertension.

In a subsequent article by Afridi et al. [329], the association between exposure to toxic elements through cigarette smoking and the occurrence of hypertension in Dublin residents was analyzed. The study included analysis of As, Al, Ni, and Pb in the hair and blood of hypertensive patients, as well as in cigarette samples. The results showed significantly higher concentrations of toxic elements in patients with hypertension, particularly smokers (*p* < 0.001), and also in smokers without hypertension, compared to the control group. Remarkably, concentrations of toxic elements were two to three times higher in the scalp hair and blood samples of smokers, even those without concomitant hypertension, compared to nonsmokers.

Another Afridi et al.’s study [330] analyzed the profiles of trace elements (Zn, Cu, Mn, Se) and toxic elements (As, Cd, Hg, Pb) in the hair and blood of smoking patients with rheumatoid arthritis (RA) living in Dublin, comparing them with a healthy, non-smoking control group. Significantly higher mean concentrations of As, Cd, Hg, and Pb were observed in biological samples in patients with RA. At the same time, the concentrations of Zn, Cu, Mn, and Se were lower in these patients, with particular statistical significance in smokers (*p* < 0.001). Furthermore, smoking individuals without RA had 2- to 3-fold higher levels of four toxic elements compared to the control group.

A study by Skalny et al. [331] conducted on 344 adult women showed that smoking significantly affects the levels of essential metals and metalloids. Smokers had lower serum concentrations of Cu, Fe, and Zn, as well as lower hair Fe and Se levels, compared to nonsmokers. Smokers had increased serum concentrations of Mn, V, and Cr. Multiple regression analysis confirmed an inverse association between smoking intensity and selenium levels, and a positive correlation with chromium levels. Additionally, an inverse relationship was found between serum zinc and hair iron levels and the number of cigarettes smoked per day. These results suggest that changes in metal and metalloid metabolism may contribute to the health risks associated with smoking.

Other metallomic studies in smokers’ hair, along with the methodology and the authors’ observations, are summarized in Table 3.

## 6. Molecular Mechanism of Metal Toxicity

Among the heavy metals found in tobacco, the highest concentrations are found in Pb and Ni, while Cd and Ni pose the greatest carcinogenic risk when inhaled, which is related to their strong tendency to accumulate in the smoker’s body [345]. The following subsections will discuss the molecular mechanisms of toxicity of these metals and the observed health effects.

### 6.1. Cd Toxicity

**Figure 4:**
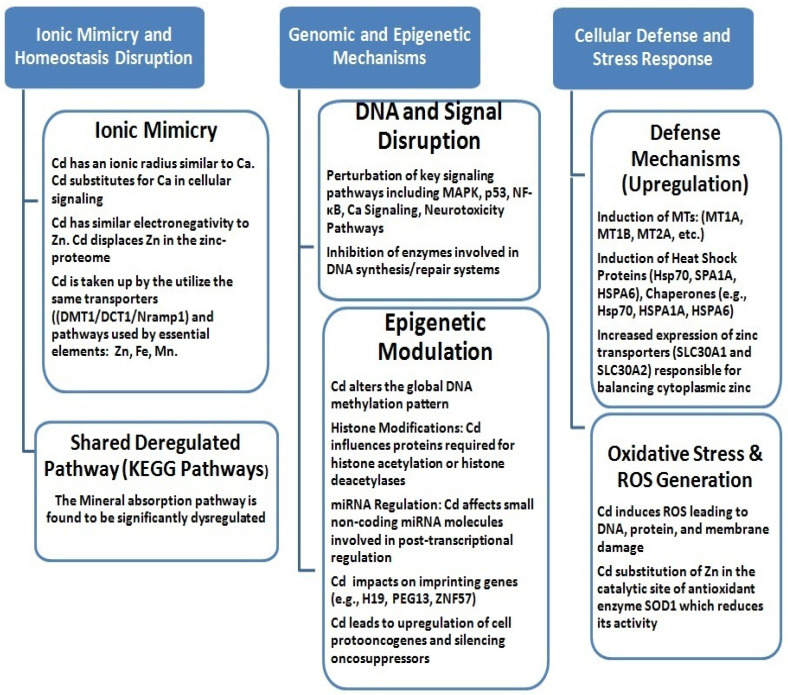
Molecular mechanisms of Cd toxicity.

The importance of Cd as a systemic toxin is underscored by two key features: its exceptionally long half-life in the human body, estimated at 10–30 years, and the definitive classification of Cd by the IARC as a Group 1 human carcinogen. This combination of persistence and carcinogenicity makes Cd a prime target for ongoing public health surveillance. To fully appreciate its clinical impact, understanding the mechanisms of toxicity is essential. Currently, the prevailing view is that Cd, through molecular/ionic mimicry, disrupts the metabolism of essential metals, leading to general metal ion dyshomeostasis, a mechanism that initiates a range of pathologies in various tissues [253,346,347]. The molecular mechanisms of Cd toxicity are shown schematically in Figure 4.

Ion mimicry, in which Cd^2+^ mimics divalent metals essential at the binding sites of various carrier proteins and/or channels, results from the ability of Cd to mimic essential divalent cations, particularly Zn^2+^, Ca^2+^, Fe^2+^, and Mn^2+^). Due to their similar ionic radius and chemical properties (ionic radius similar to Ca and similar electronegativity to Zn), Cd utilizes ion channels (especially calcium channels) and divalent metal transporter 1 (DMT1/DCT1/Nramp1) for these essential metals, leading to widespread cellular metal dyshomeostasis [348,349]. This disruption is so crucial to its toxic effects that the mineral uptake pathway has been identified as a single KEGG pathway, significantly dysregulated in lung, liver, and neuronal cell models, indicating its common initial factor in Cd-induced cell damage.

The toxic effects of Cd also include the induction of oxidative stress and the generation of reactive oxygen species (ROS), inducing metabolic disturbances in cells and, subsequently, functional disturbances in many organs. Cd-induced ROS production in neuronal models is caused, among other things, by a reduction in enzyme activity due to interactions with Zn, Cu, Fe, Mg, Ca, and Se ions. Cd exposure affects SOD1 due to the replacement of Zn by Cd, CAT, and glutathione peroxidase and reductase (GSHPx and GSHR), contributing to the induction of oxidative stress and, consequently, peroxidative damage to cell membranes [350]. In response to oxidative stress and the resulting proteotoxicity, cellular systems demonstrate the induction of metallothioneins (MTs) and heat shock proteins (Hsp70). MTs are small, cysteine-rich proteins crucial for the chelation of metal ions (Zn and Cu) and the sequestration of toxic elements such as Cd. Toxicogenomic studies have found that genes encoding MT (including MT1A, MT1B, MT1E, MT2A) are strongly regulated (usually upregulated) in all cell models studied (liver, lung, and neuronal), confirming the role of MT as a primary line of defense.

The work of Forcella et al. [346] describes a toxicogenomic study comparing how Cd exposure affects gene expression in three human cell models: lung (A549), liver (HepG2), and neuronal (SH-SY-5Y), identifying both common and tissue-specific molecular mechanisms of toxicity, such as dysregulation of metal homeostasis (mineral absorption pathway), increased expression of metallothioneins, activation of oncogenes and apoptotic pathways. According to the analysis of Forcella et al. [346], Cd alters the expression of various genes, e.g., 32 genes upregulated in all three models studied, involved in tumor promotion, proliferation, and invasiveness (e.g., BATAF, CREB5, LAMB3). Analysis of the common features of DEGs in three cell models revealed that only four genes were downregulated in all cell models exposed to Cd: AMDHD1, encoding a protein protective against breast cancer [351,352], KAZALD1, associated with hypermethylation in malignant pleural mesothelioma [353,354], KLHDC9, associated with lung adenocarcinoma [355], and RAB26, associated with important regulators of vesicular fusion and transport [356], and breast cancer cell migration and invasion [351]. Cd negatively affects signaling pathways, including mitogen-activated protein kinase (MAPK), NF-κB, and the p53 pathway. Deregulation of the p53 pathway is observed in neuronal cells. Activation of p38-MAPK by Cd leads to increased expression of genes associated with inflammation and cell death. Cd interferes with autophagy, a self-degradation process crucial for the elimination of damaged organelles. It can act both protectively and promote cell death (apoptosis), depending on the level of autophagy. Cd-induced increases in intracellular Cd lead to the induction of ROS, which in turn initiates apoptosis. However, the authors of the study emphasize the rather ambiguous role of autophagy. They concluded that Cd can act as both a protector and a promoter of cell death (apoptosis) by interfering with the autophagy (self-degradation) process. This conflicting effect on cell fate is determined by the “appropriate level of autophagy needed to maintain cell survival” [253].

**Figure 5:**
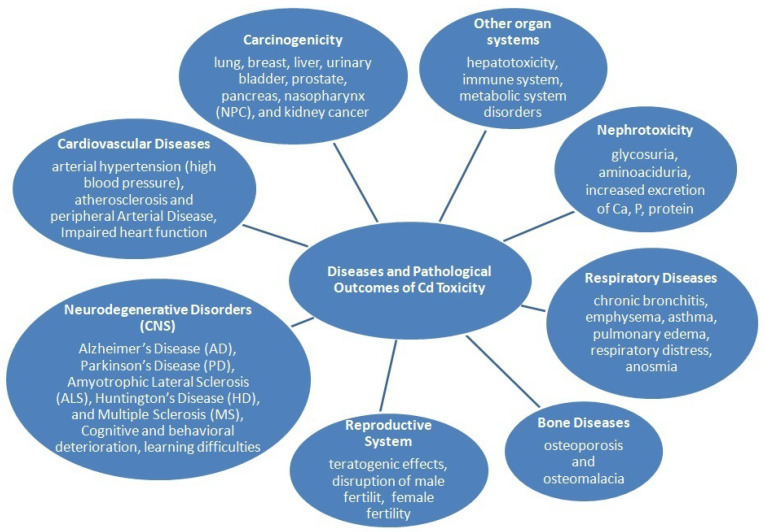
Cd toxicity—health outcomes.

Although Cd is recognized as an epigenetic modulator (DNA methylation, regulation of imprinting-related genes (e.g., H19 and PEG13), there is considerable uncertainty regarding its effect on global DNA methylation. Short-term exposure to Cd has been shown to reduce DNA methylation, whereas chronic exposure can lead to hypermethylation (due to the induction of DNA methyltransferase-1–DNMT1) [346,357]. The health effects of Cd-induced toxicity and disease can be observed in various body systems, mainly in the lungs and kidneys (Figure 5).

The kidneys are the primary target organ and the most sensitive to Cd toxicity. Cd accumulates in the proximal tubular epithelial cells, impairing tubular and glomerular function and leading to renal failure [347,358,359]. MTs sequester Cd as a defense mechanism. The CdMT complex is released into the blood and then into the kidneys, where it is reabsorbed by the proximal tubular cells. Depletion of MT stores in the kidneys causes unbound Cd to damage the proximal tubular epithelial cells and leads to toxic nephropathy and reduced capacity for nutrient reabsorption, glycosuria, aminoaciduria, and increased excretion of low molecular weight (LMW) proteins, Ca, and P [347,360,361]. Because Cd interferes with the metabolism of Ca, Mg, Fe, Zn, and Cu, it leads to skeletal disorders such as bone demineralization, osteomalacia, and osteoporosis. This effect is caused by competitive displacement of Ca ions and inhibition of the enzyme 1-hydroxycholecalciferol hydroxylase, necessary for converting vitamin D to its active form in the kidneys, which limits Ca absorption from the intestines [347].

Deterioration of renal function in smokers has been confirmed in recent years. 10,267 individuals over the age of 20 from the NHANES were examined for serum cotinine concentration and glomerular filtration rate (eGFR). A negative correlation was found between serum cotinine concentration and eGFR [362]. Long-term inhalation exposure to Cd from tobacco smoking can severely damage the respiratory system and lead to pulmonary edema, chronic bronchitis, emphysema, and abnormal lung function, and even complete loss of the sense of smell (anosmia) [363]. Cd exposure is associated with cancers in various organs: lung, breast, liver, bladder, prostate, pancreas, and nasopharynx. Cd toxicity at the cellular level promotes oncogenesis through the induction of oxidative stress, epigenetic effects, and the activation of genes associated with proliferation and metastasis (e.g., BATAF, CREB5, LAMB3) [253,346,347,364,365,366].

Cd has been implicated in the etiopathogenesis of neurodegenerative diseases, including AD, PD, amyotrophic lateral sclerosis (ALS), and multiple sclerosis (MS) [367,368,369,370]. Higher Cd concentrations in children correlate with neurological disorders, learning disabilities, and cognitive decline [371]. Neurotoxicity is associated with Cd-induced oxidative stress and impaired Ca/Zn signaling. Meysami et al. studied 10,134 participants and performed MRI on smokers and nonsmokers and compared gray and white matter volume normalized to total intracranial volume using a two-tailed *t*-test. Smokers had lower normalized gray matter volumes (t = −7.806 × 10^0^, *p* = 6.508 × 10^−15^) and white matter volumes (t = −7.374 × 10^0^, *p* = 1.791 × 10^−13^) compared to nonsmokers. Long-term smoking has been shown to cause volume loss in areas such as total gray matter volume, total white matter volume, temporal lobe, parietal lobe, hippocampus, precuneus, and posterior cingulate gyrus [371]. Chronic exposure to Cd can lead to hypertension, atherosclerosis, and impaired heart function. Smokers have higher blood Cd levels, which correlate with an increased risk of developing atherosclerosis and peripheral arterial disease (PAD) [372,373]. The study by Li et al. [374] The impact of tobacco smoking (via Cd) on cardiovascular disease was studied over almost 20 years in a Swedish population-based cohort of 4304 men and women. The measured blood Cd concentration was 0.43 μg L^−1^ (median 0.24 μg L^−1^) and increased with smoking. Shorter survival and a higher incidence of cardiovascular disease were observed in smokers.

Cd can cross the placenta, exerting teratogenic effects [375,376]. In women: Cd concentrations in blood, urine, and kidneys are often higher in women than in men, which may be related to iron deficiency. Cd has also been associated with impaired fertility and hormonal disruption [377]. In men, Cd toxicity impairs testicular function (necrosis of the seminiferous tubules, damage to Leydig and Sertoli cells) and disrupts testosterone synthesis and spermatogenesis. The number of normal sperm has been found to decrease with increasing blood Cd concentrations [378]. Cigarette smoking has a clear and significant negative impact on the reproductive system of both men and women, leading to a wide range of problems related to fertility and pregnancy outcomes [379].

### 6.2. Pb Toxicity

**Figure 6:**
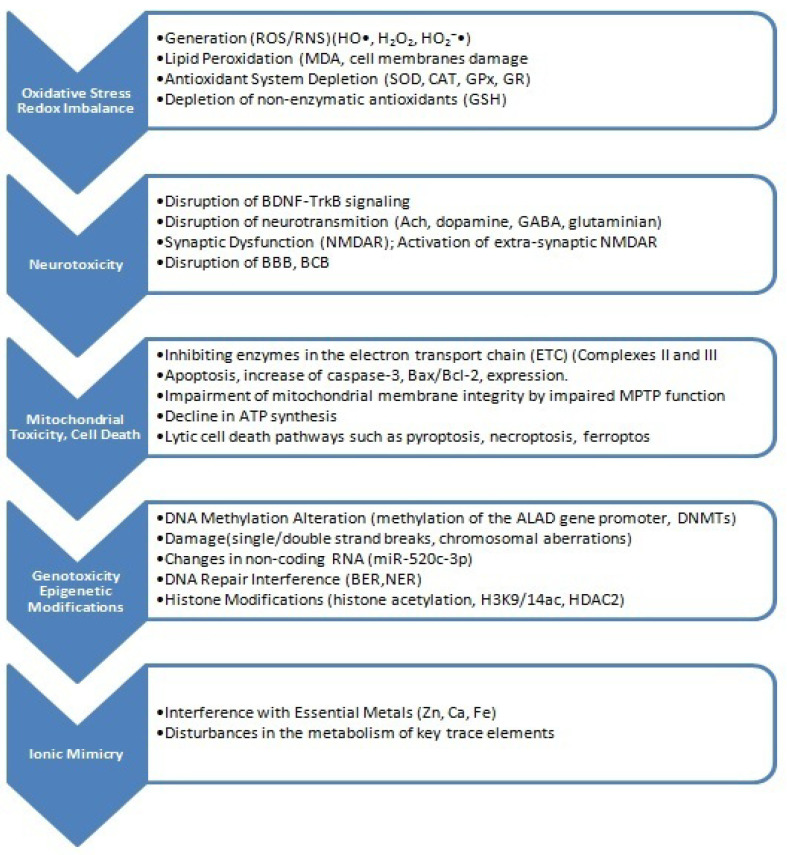
Molecular mechanisms of Pb toxicity.

Lead (Pb) is a highly toxic metal that has no known biological function in the body and affects almost all organ systems (Figure 6).

The toxic effects of Pb are multifactorial and include redox imbalance and the generation of oxidative stress through the overproduction of ROS (singlet oxygen, hydroxyl radicals (HO^•^), hydrogen peroxide (H_2_O_2_), superoxide anions, and hydroperoxides (HO_2_^−•^), which are considered the main mechanism of lead toxicity [379,380]. Pb also causes hemoglobin oxidation, which directly leads to erythrocyte hemolysis. This occurs due to inhibition of the ALAD enzyme (δ-aminolevulinic acid dehydratase), resulting in an increase in the concentration of the substrate (δ-aminolevulinic acid) ALA in both blood and urine. These elevated levels of ALA generate hydrogen peroxide and superoxide radicals and also interact with oxyhemoglobin to produce hydroxyl radicals [381]. Pb’s inhibition of the antioxidant system results from its high affinity for the sulfhydryl (-SH) groups of proteins, which inactivates antioxidant enzymes such as SOD, CAT, GPx, and GR [382,383,384]. Pb can also substitute for Zn and Cu ions, which are cofactors for these enzymes [385,386]. Pb binds to the thiol groups of glutathione (GSH), reducing its levels, which interferes with the recycling of oxidized glutathione (GSSG) to the reduced form (GSH) and depletes antioxidant reserves [385]. Lipid peroxidation causes the generation of ROS. As a result of ROS attack on polyunsaturated fatty acids in cell membranes, lipid oxidation products such as malondialdehyde (MDA) and 4-hydroxynonenal (4-HNE) are formed, which are highly reactive and can damage other macromolecules, including DNA [383,387].

Another mechanism responsible for Pb toxicity is ion mimicry and interference with essential metal metabolism. Pb exerts its toxic effects by mimicking essential divalent cations, primarily Ca, Zn, and Fe [380,388]. By mimicking Ca, Pb enters cells, including neurons, through voltage-gated calcium channels and the N-methyl-D-aspartate receptor (NMDA), disrupting calcium homeostasis, leading to increased intracellular calcium concentration. Mitochondrial dysfunction has been demonstrated to result in neuronal death [389,390]. Pb competes for Ca^2+^ binding sites on key proteins such as calmodulin (CaM), protein kinase C (PKC), and synaptic proteins and troponin C. Activation of PKC by picomolar concentrations of Pb^2+^ can lead to aberrant activation of transcription factors and act as a tumor promoter [391,392]. Interference with Zn^2+^ and Fe^2+^ is also possible. Pb^2+^ substitutes for Zn^2+^ in zinc finger proteins (e.g., transcription factors Sp1 and Egr-1), disrupting the regulation of gene expression (e.g., in the developing brain) [393,394]. Pb^2+^ can substitute for Fe^2+^ in divalent metal transporter 1 (DMT1), facilitating its own transport [395]. Recent studies indicate that lead exposure increases Fe^2+^ levels in the rat brain, which is associated with reduced expression of ferroportin 1 (FP1), a key Fe export protein [396].

Another mechanism responsible for Pb toxicity is mitochondrial toxicity and apoptosis. Mitochondria are considered the primary target of Pb toxicity, leading to energy-related disruptions. Pb^2+^ ions accumulate in the mitochondrial matrix, where they inhibit the activity of respiratory chain enzymes (particularly complexes II and III), leading to dysfunctional oxidative phosphorylation and decreased ATP synthesis [386]. Pb induces programmed cell death (apoptosis) in neurons and hepatocytes [379]. The mechanisms responsible for this process include oxidative stress, increased intracellular Ca^2+^ levels, and an imbalance of pro- and anti-apoptotic proteins (e.g., increased p53 and Bax expression, altered Bax/Bcl-2 ratio, and cytochrome C release) [383]. Recent studies indicate that Pb also triggers non-apoptotic cell death pathways, such as ferroptosis (via increased free iron and downregulation of GPX4) and necroptosis [396].

There is scientific evidence that Pb ions induces autophagy/mitophagy (selective degradation of damaged mitochondria), which is an adaptive mechanism but can also lead to cell death. This mechanism involves regulation of mitochondrial dynamics proteins (MFF, Drp1, MFN) and acceleration of mitophagy via the PINK1/Parkin pathway [386,396]. Pb is classified by the IARC as a probable human carcinogen (Group 2A/2B) and is responsible for genotoxicity and epigenetic changes. Pb damages DNA directly (by binding to oxygen atoms in nucleic acids or nitrogen atoms in DNA bases, e.g., the formation of Pb^2+^-DNA complexes in the minor groove) or indirectly through ROS and oxidized lipids (causing strand breaks, chromosomal aberrations, and the formation of 8-hydroxy-2′-deoxyguanosine (8-OHdG)) [379]. Pb disrupts DNA repair mechanisms, including base excision repair (BER) and nucleotide excision repair (NER) [397,398].

A key recent development has been the evidence that Pb^2+^ inhibits the activity of apurinic/apyrimidinic endonuclease 1 (APE1/Ref-1), an enzyme critical for DNA repair, by directly binding to divalent cation sites [399,400]. Lead induces telomere instability in human cells, which is associated with the formation of gammaH2AX foci (indicating double-stranded DNA breaks) and the stabilization of G-quadruplexes by Pb^2+^ ions, leading to telomere shortening [401]. Pb also modulates gene expression through epigenetic mechanisms [402]. Pb toxicity alters DNA methylation status, for example, by increasing methylation of repeat element promoters (LINE-1) or the ALAD gene promoter, which represses its transcription [397,398,399,400,401,402,403,404,405].

Schneider et al. demonstrated that Pb also affects methyltransferases (DNMT1, DNMT3a) and MeCP2 (methyl cytosine-binding protein) and posttranslational histone modifications (e.g., increased histone acetylation) in the hippocampus [406]. Pb is toxic to the central nervous system. Specific mechanisms, such as disruption of neurotransmission through effects on neurotransmitters and their receptors, are responsible for Pb’s neurotoxicity [379,388]. For example, Pb^2+^ is a potent, noncompetitive antagonist of the N-methyl-D-aspartate receptor (NMDAR), binding at the Zn^2+^ site [407,408,409]. Recent reports indicate changes in the expression of NMDAR subunits (e.g., an increased proportion of NR2B subunits) and the activation of extrasynaptic NMDAR receptors, which leads to the shutdown of the CREB phosphorylation pathway (a transcription factor crucial for memory and synaptic plasticity) [410,411]. Pb^2+^ neurotoxicity induces hyperphosphorylation of tau protein (e.g., at positions Ser 199/202 and Thr 231), leading to microtubule damage and disintegration [412]. Energy deficits in neurons through the inhibition of key glycolytic enzymes (hexokinase and pyruvate dehydrogenase complex (PDHc)) in the brain, which leads to a longer-term increased risk of neurodegenerative diseases [382].

In recent years, attention has been drawn to the fact that Pb^2+^ poisoning induces neuroinflammation through the activation of microglia and astroglia. This leads to increased levels of proinflammatory cytokines (IL-1β, IL-6, IL-8, tumor necrosis factor (TNFα)) and activation of signaling pathways such as NF-κB, JNK/MAPK kinases, and nuclear factor erythroid 2-related kinase (Nrf-2) [413,414]. In lung cell cultures, lead acetate has been observed to induce activation of the epidermal growth factor receptor (EGFR), autophosphorylation of Src family kinases (SFKs), and an increase in Ras-GTP [415,416].

The main toxic effect of Pb^2+^ in blood is red blood cell damage. Pb inhibits heme synthesis enzymes (ALAD, ferrochelatase) and leads to microcytic and hypochromic anemia [389]. Dysfunction of blood cell metabolism has also been confirmed by inhibition of glycolysis (e.g., glyceraldehyde-3-phosphate dehydrogenase, pyruvate kinase) and the pentose phosphate pathway, which leads to decreased ATP concentration in erythrocytes [417], inducing phosphatidylserine (PS) translocation to the outer membrane of erythrocytes, and accelerating eryptosis (a type of red blood cell death), which may be related to procoagulant activity and anemia [418]. Pb immunotoxicity manifests itself through a shift in the immune response towards Th2 lymphocytes, resulting in increased IgE and IL-4 concentrations [419].

The molecular mechanisms of Pb toxicity vary, resulting in diverse biological effects of exposure. Exposure to Pb, even at low levels, leads to serious health problems, especially in children, and an increased risk of cancer and cognitive impairment. Neurotoxicity manifests itself in the form of cognitive and behavioral disorders, i.e., lower intelligence quotient (IQ) and cognitive impairment, learning difficulties, problems with short-term memory and attention (attention deficit/ADHD), abnormal behavior, irritability, aggression (in children), increased risk of psychological disorders (depression, anxiety) and crime (in adulthood, related to childhood exposure [379,380,385,402,415,420,421]. High exposure to Pb may lead to encephalopathy (progressive brain degeneration, brain edema), convulsions, coma, paralysis, ataxia (lack of coordination) as a result of structural and molecular damage, i.e., blood-brain barrier (BBB), hypomyelination and demyelination (glial cell damage), neurotransmission disorders (e.g., blocking of NMDA receptors) [379,380]. Exposure to Pb affects the hematopoietic system, leading to anemia, which manifests itself as changes in blood parameters: decreased hemoglobin (Hb), hematocrit (Hct), and red blood cell (RBC) counts, as well as a potential increase in white blood cells (WBC) and platelets (PLT) [380,382].

The kidneys are at risk of damage because Pb is excreted and accumulates within them. Acute nephropathy is characterized by impaired tubular transport, degeneration of the tubular epithelium, and the presence of inclusion bodies. It can lead to Fanconi syndrome [380,382]. Chronic kidney disease can lead to irreversible functional and morphological changes, including renal failure, hypertension, and hyperuricemia (gout) due to glomerular damage and increased urea and creatinine levels [382,422].

Pb It is associated with cardiovascular diseases such as hypertension (often at low exposure levels), increased risk of stroke, coronary artery disease, coronary artery disease and arrhythmia, atherosclerosis (atherosclerosis) and endotheliitis, and impaired cardiomyocyte contractility [380,382,423].

Pb toxicity affects reproductive function in both men and women. In men: Reduced sperm count and motility, abnormal morphology, chromosomal damage, infertility, and changes in testosterone levels. In women: Infertility, miscarriages, premature rupture of membranes, premature birth, and gestational hypertension. Pb has been identified as an endocrine disruptor [424,425,426,427,428].

The liver is one of the main organs affected by Pb toxicity. Exposure can lead to nonalcoholic fatty liver disease (NAFLD) and elevated enzymes. Hepatic enzymes (e.g., transaminases, bilirubin). Necrosis and fibrosis are possible [429,430,431]. Bone: Pb is stored in bone (85–95% in adults) for decades. It can replace Ca in hydroxyapatite crystals, leading to decreased bone mineral density (BMD) and increased susceptibility to fractures [432,433,434].

Pb toxicity increases the risk of genotoxicity and cancer development. IARC classification: The IARC classifies Pb as a probable human carcinogen (Group 2A). HHS classification: The US Department of Health and Human Services (HHS) considers Pb to be a substance that may reasonably be expected to be carcinogenic to humans. Epidemiological studies have shown an association between exposure to Pb (particularly occupational exposure, but also in the general population) and the development of several types of cancer, including lung cancer, kidney cancer, brain cancer, including meningioma, other cancers of the esophagus, stomach, rectum, Hodgkin’s lymphoma, and acute myeloid leukemia [421]. The mechanisms responsible for Pb genotoxicity include oxidative stress (generates ROS, inhibition of enzymes (SOD), (CAT) and (GPx), lipid peroxidation), DNA damage (DNA strand breaks (single or double), chromosomal aberrations and genome instability, induction of apoptosis, ion mimicry towards divalent cations, (Ca^2+^, Zn^2+^ and Fe^2+^), mitochondrial dysfunction, immunotoxicity and increased risk of autoimmune diseases [382,435].

### 6.3. Ni Toxicity

Ni toxicity in smokers is mainly due to inhalation of tobacco smoke, which is a significant source of exposure to this element [223]. Ni in tobacco smoke can occur in the gaseous phase or as particles. Of particular toxicological importance is Ni carbonyl (Ni(CO)4), which, due to its lipid solubility, rapidly penetrates the alveolar barrier into the bloodstream after inhalation [436]. Studies by Stojanović et al. showed high Ni content in both tobacco (2.20–4.91 mg/kg) and cigarettes (2.32–4.20 mg/kg), regardless of their type and origin; one cigarette contains from 1.1 to 3.1 g of Ni. Biomonitoring of Ni concentrations in body fluids showed that urinary nickel concentrations in smokers were significantly higher (median 1.20 μg/L) compared to nonsmokers (median 0.50 μg/L) (*p* < 0.05). Blood Ni concentrations in smokers (median 0.07 μg/L) were higher than in nonsmokers (median 0.06 μg/L), although this difference was not statistically significant (*p* > 0.05) [223]. High exposure to carcinogenic Ni in smokers increases health risks. Inhalation of Ni is the main route leading to toxicity in the respiratory tract, lungs, and immune system. Ni exposure can cause lung and nasal cancer, pulmonary fibrosis, and cardiovascular and renal diseases, among others [437,438,439]. A higher incidence of malignant diseases has also been observed in smokers exposed to Ni [440]. The mechanism of Ni toxicity is complex, and although not yet fully understood, research indicates the key role of several molecular mechanisms, including oxidative stress and mitochondrial dysfunction [441]. Mitochondrial damage by Ni leads to impaired mitochondrial membrane potential, reduced ATP concentration, and mitochondrial DNA destruction [442]. In turn, mitochondrial damage disrupts the electron transport chain, leading to increased production of ROS and increased oxidative stress [443]. Exposure to Ni nanoparticles increases the levels of ROS, nitric oxide (NO), and malondialdehyde (MDA—a marker of lipid peroxidation), while simultaneously reducing the activity of antioxidant enzymes such as SOD and CAT [444]. Ni is classified as a carcinogen (Ni compounds as Group 1). The carcinogenic potential of Ni is related to its ability to induce epigenetic changes [445]. Ni is capable of replacing other metal ions in enzymes, e.g., the iron ion at the catalytic sites of iron- and 2-oxoglutarate-dependent dioxygenases. These enzymes, such as the histone demethylase JMJD1A and the DNA repair enzyme ABH2, are targets of Ni [441].

Ni exposure leads to changes in the epigenetic landscape, including DNA hypermethylation, histone modifications (e.g., H3S10 hyperphosphorylation, H3K4 hypermethylation, H2A and H2B hyperubiquitination), and interference with the microRNA (miRNA) network [446,447,448]. Ni ions are capable of inducing heterochromatization by binding to DNA-histone complexes and initiating chromatin condensation. This can lead to gene silencing (e.g., tumor suppressor genes), cell transformation, and ontogeny [449].

Ni^2+^ ions can induce apoptosis by activating caspase-dependent pathways: Intrinsic (mitochondrial) pathway: Ni^2+^ facilitates the release of cytochrome C (Cyt C) from mitochondria into the cytosol. Cyt C activates procaspase-9, which in turn activates caspases-3, -6, and -7. Extrinsic pathway: At the cell surface, Ni^2+^ promotes the interaction between Fas (First Apoptotic Signal) and FasL (Fas Ligand), leading to the activation of caspase-8 and -10. These caspases activate caspases-3, -6, and -7. In both pathways, effector caspases (mainly-3, -6, and -7) act on PARP, leading to apoptosis [441].

Ni is responsible for enzymatic inhibition by binding to specific amino acid residues in the active site (such as cysteine, histidine, glutamate, and lysine), blocking catalytic activity; binding to secondary (allosteric) sites, e.g., binding to ATP:Cob(I)alamin adenosyltransferase, the activity of which decreases by approximately 50% following Ni exposure [450,451,452].

Ni compounds have embryotoxic and teratogenic properties, as demonstrated in numerous experimental studies [441]. Leonard and colleagues, who confirmed the strong teratogenic effects of nickel compounds, hypothesized that the prenatal effects of Ni may be partially due to changes in mitosis leading to cell death [453,454].

**Figure 7:**
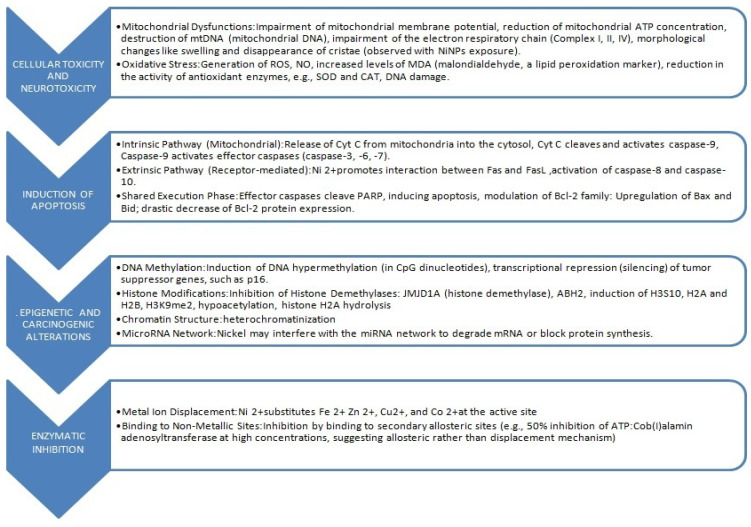
Molecular mechanisms of Ni toxicity.

Ni is a potent allergen, leading to the production of cytokines or chemokines [441]. The reaction involves the activation of antigen-presenting cells (APCs) and T lymphocytes in complex immune responses. Activated APCs migrate to lymph nodes, where they present Ni (allergen/hapten) to naive CD4+ T lymphocytes. Upon re-exposure to the same hapten, T cells are stimulated and proliferate, entering the bloodstream and producing visible hypersensitivity symptoms 48–72 h after allergen exposure. After repeated exposures, T cell clones reach a “threshold” and a skin rash develops, which can manifest as acute, subacute, or chronic eczema-like patches [455,456]. The pathogenesis and precise mechanisms of the allergic response to Ni remain incompletely understood. The molecular mechanisms of Ni toxicity are schematically shown in Figure 7.

## 7. Summary and Future Directions

Tobacco (*Nicotiana tobacum* L.), used in cigarette production, contains, among more than 7000 identified chemical compounds [123,457], heavy metals such as Pb, Cr, Ni, As, and Cd [345]. Many of them are carcinogenic and mutagenic substances [457]. The metal content in tobacco ranges from less than 1 μg/g (Co, Cd, Pb, As, and Tl) to several hundred μg/g (Al, Mn, and Ba) [458]. The toxicity of metals and their bioaccumulative properties pose a serious long-term health risk and contribute to the development of tobacco-related diseases, particularly respiratory diseases (emphysema, chronic bronchitis, chronic obstructive pulmonary disease, and lung cancer) [118,459,460,461,462,463,464,465,466,467,468,469,470]. The number of deaths due to tobacco-related diseases is predicted to increase to eight million by 2030 [7]. Numerous studies are being conducted to quantify these metals in cigarettes, tissues, and body fluids of tobacco smokers, and to examine the correlation between the accumulation of toxic metals and health risk assessments. There is no doubt that toxins from cigarettes originate from the tobacco growing site and the technological processes used in its production. Until now, the ability of plants such as *Nicotiana tabacum* to accumulate metals has been primarily exploited for the removal of metal contaminants from soil (bioremediation). To ensure that tobacco cultivation for cigarette production does not pose a health risk, it must meet ecological requirements. Changing cigarette production technology could also improve the situation.

It has been proven that the group of elements that accumulate in tissues as a result of chronic smoking primarily includes Pb, Al, Cr, Ni, As, and Cd. Despite existing evidence of metal accumulation in the human body as a result of cigarette smoking and passive smoking, there are still few studies demonstrating the general distribution of toxins and the organs that preferentially accumulate them. We know that smoking disrupts mineral homeostasis, but further research is needed on interelemental correlations that would allow for preventive supplementation and potentially even therapy.

Currently, thanks to the development of analytical techniques, it is possible to analyze multi-element elements present in trace and ultra-trace amounts even in small sample masses. Therefore, attention has recently drawn to the increased content of rare earth elements in the tissues of smokers, whose role in the body and toxicity are poorly understood. The main analytical challenge is lowering detection limits for ultra-trace elements, alongside significant variations in total metal concentrations in biological samples.

The lack of consistency in the results of metallomic studies conducted by different teams may be due to the variety of methodologies used, but also to objective factors such as the origin of the tobacco. The selection of the control and treatment groups took into account lifestyle, diet, chronic diseases, and medications taken, which affect the body’s mineral status and may mask or synergize with smoking in the processes of metal accumulation in smokers. For example, Mortada et al. [471] reported blood Pb levels of 101.6 + 30.9 mg L^−1^ in nonsmokers and 143.7 + 33.8 mg L^−1^ in smokers, while Satarug et al. [472] reported much lower serum Pb levels of 4.2 + 5.4 mg L^−1^ in nonsmokers and 9.0 + 12 mg L^−1^ in smokers.

Based on a review of recent literature, only one proposal can be found for eliminating toxic elements accumulated as a result of smoking. Zn supplementation has been found to prevent Cd accumulation. In the case of plants, Mg biofortification has been observed to protect tobacco plants from Al accumulation. The idea of searching for interelemental correlations may be a useful strategy in further research, especially since toxicogenomic studies indicate the mechanism of molecular/ionic mimicry as a common process initiating other pathologies.

## Figures and Tables

**Table 1:** Metal content in tobacco products.

Tobacco Product	Metal	Average Metal Concentration	Ref.
commercial moist snuff and Alaskan iqmik	As, Ba, Be, Cd, Cr, Co, Pb, Ni	0.23 ± 0.06 µg/g (As); 1.40 ± 0.31 µg/g (Cd); 0.45 ± 0.13 µg/g (Pb); 2.28 ± 0.36 µg/g (Ni)	[173]
Tobacco from U.S. Cigarettes (n = 50)	As, Be, Cd, Cr, Co, Pb, Mn, Hg, Ni	Mn 131–245 µg/g; Cr 1.4–3.2 µg/g; Co 0.44–1.11 µg/g; Ni 2.1–3.9 µg/g; Cd 1.0–1.7 µg/g; Pb 0.60–1.16 µg/g; As 0.29 µg/g; Be 0.031 µg/g	[138]
cigarette filler and ash from 36 brands retailed in Saudi Arabia	Cr, Cd, Cu, Fe, Pb, Mn, Zn	0.66 mg/kg (Cr), 0.09 mg/kg (Cd), 2.61 mg/kg (Cu), 245.55 mg/kg (Fe), 0.38 mg/kg (Pb), 3.985 mg/kg (Mn); 1.64 mg/kg (Zn)	[174]
Tobacco Products Available in the United States	Al	cigarette tobacco 0.719 ± 0.157 μg/mg; smokeless tobacco 0.408 ± 0.104 μg/mg; little cigar 1.20 ± 0.30 μg/mg; pipe/roll your own 1.05 ± 0.31 μg/mg	[175]
tobacco and cannabis	Al	Bangladeshi tobacco 2.3–3.7 mg Al/g; Rothmans 0.6–1.5 mg Al/g; natural tobacco 1.2–2.0 mg Al/g; medium tobacco 0.8–1.4 mg Al/g; cannabis (2 types) 0.1–0.4 mg Al/g; cannabinoid extract (THC) 0.1–0.4 mg Al/g	[176]
tobacco, filter and ash of illicit brands cigarettes marketed in Brazil	Cr	0.32–0.80 µg/ashes 0.96 to 3.85 μg/cigarette for tobacco	[177]
7 commercially available branded cigarettes purchased from local market of Dublin (Ireland)	As, Al, Cd, Ni, Hg, and Pb	9.55–12.4 ng/cigarette, 0.432–0.727 μg/cigarette, 360–496 μg/cigarette, 1.70–2.12 μg/cigarette, 0.715–1.52 μg/cigarette, 0.378–1.16 μg/cigarette	[178]
packs of 20 cigarette brands were purchased from retail outlets at Oporto, Portugal	Co, Cd, Pb, As, Tl, Al, Mn, Ba	from less than1 μg/g (Co, Cd, Pb, As i Tl) to several hundreds of μg/g (Al, Mn i Ba)	[156]
different components of Pakistani local branded and imported cigarettes	Al, Cd, Ni, Pb	97.3–99.0%, and 15.0–31.3% of total metal contents were observed for Al and Cd in ash, respectively	[179]

**Table 2:** Studies on the metal content in samples taken from the respiratory tract of smokers.

Tissue	Chemical Element	Observation	Ref.
bronchoalveolar space	extracellular Cd	Cd concentrations increased in cell-free BAL fluid of smokers compared to nonsmokers (n = 19–29; *p* < 0.001)	[242]
SELF fluid	inhalation bioaccessibility of Cd, Cr, Ni, Zn	The percentage inhalation bioaccessibility ranged between 20.8–59.8% (Cd), 3.3–8.1% (Cr), 21.7–48.8% (Ni) and 7.6–12.5% (Zn).	[245]
bronchial epithelium	Ca^2+^ signaling	Ca^2+^ signaling is impaired in smoker epithelia	[246]
bronchial biopsy specimens	Al potroom emissions	airway inflammation is a central feature of potroom asthma	[247]
bronchial autopsy tissue	41 elements	significantly higher concentrations of Al in bronchial and lung of smokers	[244]
non-neoplastic lung injury	Fe	disruption of iron homeostasis in cells after exposure to CSP	[248]
lung	Fe	increased extracellular Fe from AMs is a potential source of oxidative damage and inflammation in the lung following CS exposure	[249]
lung samples of controls and COPD patients	Cd, Mn	the accumulation of Cd, Mn suggests a role for these metals in the pathogenesis of COPD	[241]
human lung tissue	Cr, Cd	concentrations of about 4.3 mg/g (dry weight) were found in smokers compared to 1.3 mg/g in non smokers; increasing concentration was observed with age and smoking time	[250]

Abbreviations: bronchoalveolar lavage fluid (BAL); the Stimulated Epithelial Lung Fluid (SELF); cigarette smoke particle (CSP); alveolar macrophages (AMs); chronic obstructive pulmonary disease (COPD).

**Table 3:** Studies on the metal content in samples taken from the hair and nails of smokers.

Studied Samples	Population	Metals	Detection Method	Observation	Ref.
blood and hair	healthy adult male volunteers, smokers (29), nonsmokers volunteers (14)	Hg, Pb, Cd, As, Se, Mn, Zn, Ni, and Cr	ICP-AES	All the investigated toxic elements levels in the hair of cigarette-smoking subjects (except Hg and Zn) were significantly higher than nonsmoker subjects	[332]
hair	236 healthy people 16–75-years-of-age	Hg	ICP-MS	Hg concentration was higher in the smoking exposure group than the non-exposure group	[333]
hair	252 pregnant women	Ag, Cd, Hg, Pb)	ICP-MS	positive correlations between cotinine and Ag (r = 0.369, *p* < 0.001), Cd (r = 0.185, *p* < 0.01), Hg (r = 0.161, *p* < 0.05), and Pb (r = 0.243, *p* < 0.001); positive correlations between nicotine and Ag (r = 0.331, *p* < 0.001), Cd (r = 0.176, *p* < 0.01), and Pb (r = 0.316, *p* < 0.001)	[334]
serum and hair	344 women 20–70 years old including 199 smokers and 145 non-smoking women	Co, Cr, Cu, Fe, Mn, V, Zn, Se	ICP-MS	smoking leads to decreased serum Cu, Fe, Zn concentrations, hair Fe, Se; increased serum Mn, V, Cr; inverse relationship between smoking intensity and Se levels, and a positive correlation with serum Cr, Zn, and hair Fe levels	[331]
blood, scalp hair	smoker and nonsmoker males who have mild and severe psoriasis	Cd, Zn	AAS	increased Cd levels in hair and blood in smoking patients with psoriasis; decreased Zn concentrations in smoking patients with psoriasis; 5% decreased Zn levels in smokers; 17.8–33.3% decreased Zn levels in non-smoking patients with psoriasis; 25% lower Cd levels in the blood of smokers; two- to three-fold higher Cd concentrations in psoriasis patients	[335]
hair	95 subjects (24 males and 71 females)	Co, Cr, Cu, Li, Sr, Pb	ICP-TOF-MS	smokers’ hair contained more Cu, while the content of Pb, Cr, and Co was similar in the hair of nonsmokers and smokers. The results were not statistically significant.	[336]
blood, toenails, and hair	163 pregnant women	Pb, Cd, As, Mn	GFAAS	passive smoking significantly increased levels of Mn, Pb, Cd	[337]
hair	-	Cd, Cu, Ni, Pb and Zn	FAAS	significantly higher concentrations of Cd and Pb in male hair and smokers than those in female and non-smokers (*p* < 0.01)	[338]
blood, nasal fluid, saliva, sputum, serum, scalp hair	male smokers and nonsmokers infected with COVID-19 and from healthy men (aged 29–59 years)	Se, Hg	AAS	Se concentrations in all samples from smokers and nonsmokers with COVID-19 were lower than those of healthy smokers and nonsmokers; Hg concentrations were elevated in both smokers and nonsmokers with COVID-19	[339]
hair	821 students (433 boys and 388 girls) SHS; 48.9% of fathers who are smokers, but 25.2% of fathers smoke in front of their children.	Cr, Mn, Ni, As, Pb, Cd	ICP-MS	increasing levels of Pb and Cd in the hair of SHS children	[340]
scalp hair	various age groups (16–32 years, 33–50 years, and 51–62 years)	Zn, Fe, Cu, Cr, Cd, Ni, Pb, As	FAAS, GF AAS	the concentrations of As (0.17, 0.81, and 0.91 μg/g), Cd (2.80, 3.81, and 3.16 μg/g), Cr (4.1, 4.2, and 5.3 μg/g), Cu (20.0, 21.0, and 21.9 μg/g), Ni (3.9, 4.6, and 4.3 μg/g), Pb (4.0, 4.8, and 5.0 μg/g), and Fe (49.0, 49.0, and 59.3 μg/g) were significantly higher in smokers for various age groups respectively. The concentrations of Zn (165, 163, and 173 μg/g v) were lower in smokers. Correlation studies for male smokers show a highly positive correlation between Cr-Cd, Cr-Ni, Cu-Fe, and Ni-Zn.	[341]
scalp hair	200 pregnant and non-pregnant women (20–35 years)	Cd, Zn	AAS	The malnourished pregnant and non-pregnant smoking women group had three to four times higher levels of Cd in their scalp hair samples than those values obtained for non-smokers; the content of Zn in scalp hair samples of the reference women was ∼20% higher than the malnourished group	[342]
hair	40 female students	Mg, Cr, Co, and Zn	ICP-MS/MS	Mg, Cr, Co, and Zn concentrations were higher for the non-smoking girls; Cd concentration was higher for passive smoking; the strong positive correlation for smokers was for Ca/Cd, Al/Fe, Mg/Cr, Na/Fe, Al/Cd, and Al/Na	[343]
supragingival dental calculus	29 subjects, including nonsmokers (n = 14) and smokers (n = 15)	26 metals and metalloids	ICP-MS	The concentrations of toxic heavy metals, As (*p* < 0.05), Cd (*p* < 0.05), Pb (*p* < 0.01), Mn (*p* < 0.01), and V (*p* < 0.01), were significantly higher in smokers than in nonsmokers	[344]

Abbreviations: inductively coupled plasma mass spectrometry (ICP-MS/MS); atomic absorptions spectrometer (AAS); flame atomic absorption spectrometry (FAAS); graphite furnace atomic ab-sorption spectrometry (GF AAS); secondhand smoke exposure (SHS); inductively coupled plasma time-of-flight mass spectrometry (ICP-TOF-MS); inductively coupled plasma-atomic emission spectrometer (ICP-AES).

## Data Availability

No new data were created.

## Abbreviations

The following abbreviations are used in this manuscript:

4-HNE	4-hydroxynonenal
8-OHdG	8-hydroksy-2′-deoksyguanozyny
AAS	Atomic absorption spectrometry
AD	Alzheimer’s disease
ADHD	Attention Deficit Hyperactivity Disorder
AES	Atomic Emission spectrometry
AFM	Atomic force microscope
AFS	Atomic fluorescence spectrometry
ALA	δ-aminolevulinic acid
ALAD	δ-aminolevulinic acid dehydratase
ALS	Amyotrophic lateral sclerosis
AM	Alveolar macrophage
APC	Antigen presenting cells
APDC	ammonium pyrrolidinedithiocarbamate
APE1/Ref-1	Apurinic/apyrimidinic endonuclease 1/redox effector factor 1
ASD	Autism Spectrum Disorder
ASV	Anodic Stripping Voltammetry
BAL	Bronchoalveolar lavage fluid
BATF	Basic leucine zipper ATF-like transcription factor
Bax	Bcl-2-associated X protein
BBB	Blood-Brain Barrier
BCB	Blood-Cerebrospinal Fluid Barrier
Bcl-2	B cell lymphoma 2
BDNF	Brain-Derived Neurotrophic Factor
BER	Base excision repair
CaM	Calmodulin
CAT	Catalase
CNS	Central nervous system
COPD	Chronic Obstructive Pulmonary Disease
CREB5	cAMP responsive element binding protein 5
CRI	Cancer Risk Index
CSP	Cigarette smoke particle
CV	Cyclic voltammetry
CVAAS	Cold vapour atomic absorption spectrometry
CVG/HG	Chemical vapor or hydride generation
Cyt C	Cytochrome c
DCT1	Divalent cation transporter 1
Dios	Jodothyronine deiodinases
DL	Detection Limit
DMT	Divalent metal transporter
DNA	Deoxyribonucleic acid
DNMT	DNA methyltransferases
DPASV	Differential pulse anodic stripping voltammetry
DPV	Differential pulse voltammetry
DRC	Dynamic Reaction Cell
EDS	Energy dispersive X-ray Spectroscopy
EDXRF	Energy dispersive X-ray fluorescence
EELS	Electron Energy Loss Spectroscopy
EFTEM	Energy-Filtered Transmission Electron Microscopes
EGFR	Epidermal Growth Factor Receptor
eGFR	An estimated GFR
ELCR	Excess Lifetime Cancer Risk
EPMA	Electron probe X-ray microanalysis
ETAAS	Electrothermal atomic absorption spectrometry
EU	European Union
FAAS	Flame atomic absorption spectrometry
Fas	First apoptotic signal
FasL	Fas Ligand
FCTC	Framework Convention on Tobacco Control
FDA	U.S. Food and Drug Administration
FP	Ferroportin
GFAAS	Graphite furnace atomic absorption spectrophotometry
GFR	Glomerular Filtration Rate
GPx	Glutathione peroxidase
GSH	Glutathione
GSH-Px	Glutathione peroxidase
GSHR or GR	Glutathione reductase
GSSG	Glutathione disulfide
GTP	Guanosine-5′-triphosphate
Hb	Hemoglobin
HCL	Hollow cathode lamps
Hct	Hematocrit
HGAAS	Hydride generation atomic absorption spectroscopy
HHS	U.S. Department of Health and Human Services
HPA	The hypothalamic-pituitary-adrenal axis
HR-CS	High resolution-continuum source
IARC	International Agency for Research on Cancer
IC	Ion chromatography
ICDA	International Chromium Development Association
ICP-AES	Inductively coupled plasma-atomic emission spectrometer
ICP-MS	Inductively coupled plasma mass spectrometry
ICP-OES	Inductively coupled plasma optical emission spectrometry
ICP-QQQ-MS	Inductively Coupled Plasma–Triple Quadrupole Mass Spectrometry
ICP-TOF-MS	Inductively coupled plasma time-of-flight mass spectrometry
ICRP	International Commission on Radiological Protection
ID-ICP-MS	Isotopic dilution ICP-MS
IL	Interleukin
IMC	Imaging mass cytometry
IQ	Intelligence Quotient
ISE	Potentiometry using ion-selective electrode
IUPAC	International Union of Pure and Applied Chemistry
KEGG	Kyoto Encyclopedia of Genes and Genomes
LA	Laser Ablation
LAMB3	Laminin subunit beta 3
LCC	Lifetime Cancer Risk
LFA	Lateral flow assay technology
LIBS	Laser-Induced Breakdown Spectroscopy
LMW	Low-molecular-weight proteins
LSASV	Linear sweep anodic stripping voltammetry
MAPK	Mitogen-activated protein kinase
MAPK/JNK	mitogen-activated protein kinase/c-Jun NH2-terminal kinase
MDA	Malondialdehyde
MeCP2	Methyl-CpG binding protein 2
miRNA	microRNA
MOF	Metal-organic framework
MRI	Magnetic Resonance Imaging
MS	Multiple sclerosis
MT	Metallothionein
NAA	Neutron activation analysis
NAFLD	Non-Alcoholic Fatty Liver Disease
NanoSIMS	Nanoscale secondary ion mass spectrometry
nano-SXRF	Nano-Synchrotron X-Ray Fluorescence
NCCH	National Center for Chronic Disease Prevention and Health Promotion
NDD	Neurodevelopmental Disorders
NER	Nucleotide excision repair
NF-κB	Nuclear factor kappa-light-chain-enhancer of activated B cells
NHANES	National Health and Nutrition Examination Survey
NIST	National Institute of Standards and Technology
NMDAR	N-methyl-D-aspartate receptor
NMR	Nuclear magnetic resonance
Nramp1	Natural resistance-associated macrophage protein
Nrf-2	Nuclear factor erythroid 2-related
PD	Parkinson’s disease
PDHc	Pyruvate dehydrogenase complex
PIGE	Particle-Induced Gamma-ray Emission
PIXE	Particle-induced X-ray emission
PKC	Protein Kinase C
PLT	Platelet Count
PS	Phosphatidylserine
RA	Rheumatoid arthritis
RAAS	Renin-Angiotensin-Aldosterone System
Ras	Rat sarcoma proteins
RBC	Red Blood Cells
ROS	Reactive oxygen species
RP-IC	Reversed-phase ion interaction chromatography
SELF	Stimulated Epithelial Lung Fluid
SEM	Scanning electron microscope
SFK	Src family kinase
-SH	Sulfhydryl groups
SHS	Secondhand smoke exposure
SIA-ASV	Sequential Injection Analysis Anodic Stripping Voltammetry
SIMS	Secondary ion mass spectroscopy
SOD	Superoxide Dismutase
STEM	Scanning Transmission Electron Microscopes
SVA	Stripping voltammetry analysis
SWASV	Square wave anodic stripping voltammetry
SWV	Square wave voltammetry
SZ	Schizophrenia
TEM	Transmission electron microscopy
THS	Third-Hand Smoke
TNF	Tumor necrosis factor
TMAH	Tetramethylammonium hydroxide
TrkB	Tropomyosin receptor kinase B
TS-FF	Thermospray flame-furnace
TXRF	Total reflection X-ray fluorescence
UV	Ultraviolet
WBC	White Blood Cells
WDS	Wavelength Dispersive X-ray Spectroscopy
WHO	World Health Organization
XAS	X-ray absorption spectroscopy
XRD	X-ray diffraction analysis
XRF	X-ray fluorescence
μSR-XRF	X-ray microfluorescence analysis using synchrotron radiation
